# Pervasiveness of the IQ Rise: A Cross-Temporal Meta-Analysis

**DOI:** 10.1371/journal.pone.0014406

**Published:** 2010-12-22

**Authors:** Jakob Pietschnig, Martin Voracek, Anton K. Formann

**Affiliations:** Department of Basic Psychological Research, School of Psychology, University of Vienna, Vienna, Austria; University of Granada, Spain

## Abstract

**Background:**

Generational IQ gains in the general population (termed the Flynn effect) show an erratic pattern across different nations as well as across different domains of intelligence (fluid vs crystallized). Gains of fluid intelligence in different countries have been subject to extensive research, but less attention was directed towards gains of crystallized intelligence, probably due to evidence from the Anglo-American sphere suggesting only slight gains on this measure. In the present study, development of crystallized intelligence in the German speaking general population is assessed.

**Methodology/Principal Findings:**

To investigate whether IQ gains for crystallized intelligence are in progress in German-speaking countries, two independent meta-analyses were performed. By means of a cited reference search in ISI Web of Science, all studies citing test manuals and review articles of two widely-used salient measures of crystallized intelligence were obtained. Additionally, the electronic database for German academic theses was searched to identify unpublished studies employing these tests. All studies reporting participants mean IQ or raw scores of at least one of the two measures were included in the present analyses, yielding over 500 studies (>1,000 samples; >45,000 individuals). We found a significant positive association between years of test performance and intelligence (1971–2007) amounting to about 3.5 IQ points per decade.

**Conclusions/Significance:**

This study clearly demonstrates that crystallized IQ gains are substantial and of comparable strength as Flynn effects typically observed for measures of fluid intelligence in Central Europe. Since mean IQ was assessed in a large number of small, non-representative samples, our evidence suggests a remarkable robustness of these gains. Moreover, in both meta-analyses strength of gains was virtually identical. On the whole, results of the present study demonstrate a pervasive and generalizing Flynn effect in German-speaking countries.

## Introduction

Generational gains of intelligence in the general population are among the most intriguing findings in intelligence research. Since James R. Flynn [1], [2] was the first to systematically describe these gains, they quickly became known as the Flynn effect [3]. Many intelligence researchers subsequently directed their attention towards this astonishing phenomenon, leading to a large body of research papers examining gains in different populations, domains of intelligence, and over several time spans [4]–[8].

Investigations of the Flynn effect in different nations showed an erratic pattern of changes in intelligence gains. Whilst average IQ in France, Japan, Israel, and the Netherlands was found to rise fast, the increase of IQ in Great Britain, Ireland, New Zealand, and Australia is only moderate. There even is evidence for stagnation of the effect in Norway and Sweden and for a reversal in Denmark [2], [9]. For instance, Teasdale and Owen [5] found that Danish average intelligence test performance increased from the early 1960s, peaked in the early 1990s, and subsequently began to decrease. Therefore, it cannot be taken for granted that once-observed Flynn effects in a specific country are to remain stable.

Additionally, evidence suggests that strength of gains depends on different domains of intelligence regarding different countries. Most investigations of the Flynn effect examined changes of fluid intelligence, since gains on this domain of intelligence seem to be more pronounced [10]. There is only slight evidence for gains in crystallized intelligence in Anglo-American countries [7], while examination of intelligence test scores of samples in German-speaking countries show gains on measures of both fluid [2] and crystallized intelligence [8]. In an earlier study, Flynn reported gains of 4.8 IQ points per decade in the Vocabulary subtest on the Wechsler intelligence scale from 1954 to 1981 in West Germany [11]. Of note, according to recent findings in an investigation to the Flynn effect on vocabulary tests in an US-American sample, subjects that were under age 17 showed smaller gains (less than a forth in magnitude) compared to subjects of 17 years of age or older [12].

Another characteristic of the majority of studies on this subject is that results are based mainly on large and representative (population-based) samples, healthy male participants (e.g., from conscript mass-testing), and study periods prior to 1970. Thus, a thorough examination of recent changes of crystallized intelligence ability patterns in the general population is warranted.

Implications of the Flynn effect are crucial to intelligence test validity. They are also essential for developing appropriate guidelines for test renorming. These considerations led within the frame of the Task Force to Intelligence of the American Psychological Association to the demand of further examination and explanation of generational IQ gains [13]. By now, even the American Psychological Association's Ethical Principles of Psychologists and Code of Conduct direct attention towards the implications of test norm obsolescence [14], [15]. Similar guidelines of national professional associations have also been established, as for instance the demand of the two biggest German professional associations of psychologists to evaluate every psychometric ability measure at least every eight years with respect to test norm obsolescence [16]. But apparently, these guidelines do not reflect the practice of every-day ability assessment, as the perennial use of test measures with obsolete norms suggests [17]. All the more, this demonstrates the necessity of assessment of development of intelligence in the general population.

To contribute to this important subject, we conducted two independent cross-temporal meta-analyses [18]–[21] on two salient measures of crystallized intelligence in Austria, Germany, and Switzerland. Data of a large number of healthy and patient-based samples (>1,000 overall samples) of men and women from 1971 to 2007 were examined, thus allowing for meta-regression of assumed year of data collection on mean task performance following a novel method in respect to investigations to the Flynn effect as outlined by Voracek [8].

## Methods

### Literature Search

A cited reference search in ISI Web of Science was performed to obtain all studies citing test manuals and review articles of one of two distinct measures of crystallized intelligence [22]–[24] (Multiple-Choice Vocabulary Test [MWT]; Vocabulary Test [WST]) [22], [24]. Used search strings were: “Lehrl S*”, “Merz J* AND 1975”, and “Schmidt K* AND 1992”. Additionally, the electronic database of academic theses in German language (Datenbank deutschsprachiger Diplomarbeiten Psychologie [database of theses in German language in Psychology]) was searched to identify unpublished papers applying the MWT or WST. Identified studies were coded into categories (publication status, type of employed test, type of sample) and statistical parameters were recorded. This coding was performed twice by the same researcher to ensure reliability of category assignment. If not reported explicitly, data acquisition for respective studies was assumed as two years preceding publication of the study or one year preceding completion of unpublished work. All identifiable relevant studies up to February 2009 were included in the analyses.

### Inclusion/Exclusion Criteria

Fulfilment of three criteria was necessary for studies to be included in data analysis. First, the MWT or WST had to be administered in the study. Second, mean IQ or sufficient statistical information to calculate mean IQ (e.g., mean raw score) had to be reported. Third, data had to be original and must not have been reported previously in another paper.

### Relevant Measures

#### MWT

The MWT is widely used to assess crystallized intelligence in healthy participants and patients. In this test, which is available in two equivalent parallel versions, participants are required to indicate one actual word in presence of four distractors in each of the 37 items. Task performance in this test is to a large extent unaffected by cognitive impairment of participants, thus providing a measure of premorbid intelligence. Besides its' wide dissemination and usability for healthy participants and patients, the fact that this test has not been renormed since the initial publication made this instrument perfectly suitable for the purposes of the present study.

#### WST

The WST was developed on the basis of the MWT and is another salient measure of crystallized intelligence. Basically, this measure is characterized by the same attributes as the MWT (a widely used measure of crystallized, premorbid intelligence that has not been renormed since initial publication), but additionally is Rasch-calibrated. The validity of the Rasch model ensures both unidimensionality and favorable scale properties of the measured construct. Similar to the MWT, participants have to indicate one actual word in the presence of five distractors in each of the 42 items.

### Statistical Analyses

A particular characteristic of cross-temporal meta-analyses is that instead of considering effect sizes, average test results over time are analyzed (in the present study weighted mean IQ of samples) [20]. Thus, the association between mean sample IQ and assumed year of data collection was examined. For the MWT and the WST, two independent cross-temporal meta-analyses were performed.

In both analyses, four methods of data-analysis were applied. In all procedures, mean IQ was weighted according to sample size. First, to assess overall changes in general intelligence over time, a weighted linear meta-regression was calculated. Mean IQ of samples was entered as dependent variable and assumed year of study performance served as predictor. Second, to account for age as a moderating variable, a multiple weighted linear meta-regression was performed using mean IQ of samples as dependent variable and publication year as well as mean age of samples as predictor variables. Third, Jensen's delta IQ [10] was calculated, providing a standard measure for average changes of IQ of samples per decade. It should be noted that this method is a rather rough measure of intelligence test score changes, as it only includes numerical information of studies published in the first three (lower bound) and last three years (higher bound) of the study period. In our analyses, this meant that the lower bound comprised information of a substantially smaller number of studies than the higher bound, thus presenting a rather unstable measure. Nonetheless, we provide numerical values for this method, as it is considered a standard means of reporting in investigations of test score changes. Fourth, a meta-regression of year of assumed study performance on within-study variances were calculated to account for the possibility of decreasing population variance in intelligence as cause for intelligence test gains. As some studies did not report deviations from mean intelligence, the number of studies in these analyses was somewhat smaller.

Additionally, a weighted multiple stepwise regression of assumed year of study performance, publication language (English vs German), publication status (published vs unpublished), and sample type (patients vs normals) on mean IQ was calculated for the MWT only. Due to sample characteristics, whether or not subjects were under age 17 was not included as a possible predictor, as mean age of subjects was 17 years or higher in all samples but four. The regression model was specified to assess interactions of assumed publication year with all moderating variables, since significant interaction terms can be interpreted as differences of the slope due to moderating variables. This method was not applied for the WST because of too few observations of the respective variables (unpublished  =  0 of 164; German language  =  17 of 164).

In both meta-analyses, assumed year of performance of the first study employing the MWT [25] or the WST [26] were subtracted from assumed year of performance of all other studies, thus producing new variables containing values from 0 (first test application of the MWT in a study) to 36 (last study included) and from 0 (first test application of the WST in a study) to 11 (last study included). Data analysis was performed using the statistical software package SPSS 17.0.1 as well as the open-source software R 2.9.0. The meta-analysis was performed in accordance with the PRISMA guidelines.

### Final Sample

For the MWT, 473 studies fulfilled our inclusion criteria yielding 960 independent samples (39,429 participants). Included studies covered a time span of 37 years (publication years: 1973–2009). Since studies describing results of 60 samples did not report mean age, they had to be omitted from multiple weighted linear meta-regression. This resulted in 437 studies yielding 900 independent samples (35,592 participants; weighted mean age of samples  =  41.50, *SD*  =  14.4; means ranging from 11.2 to 87.6 years). In general, samples from individual studies were small and mainly patient-based (57.8% patients out of 39,429 participants and 57.0% patients out of 35,592 participants).

**Figure 1:**
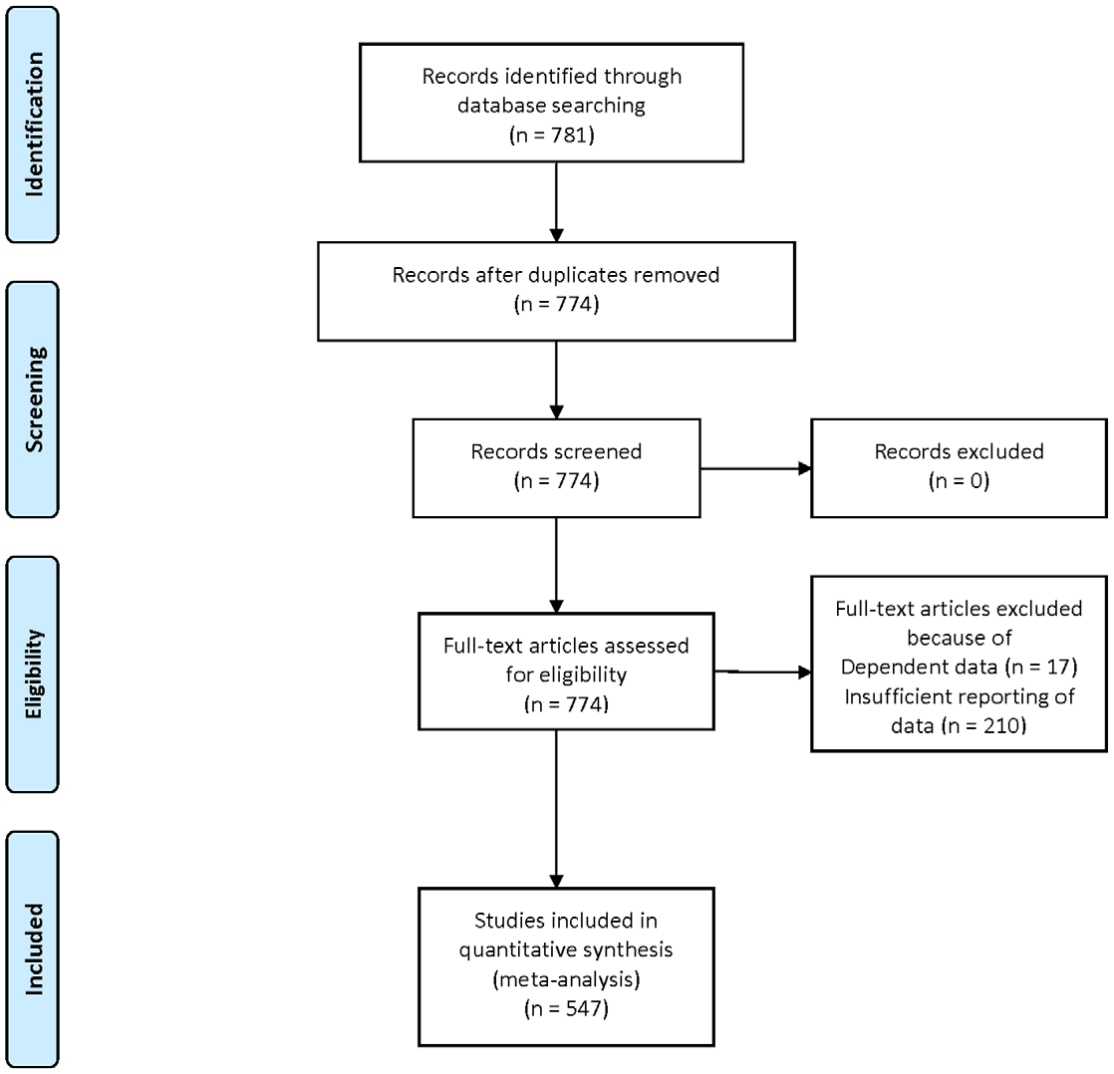
Flowchart illustrating study inclusion and exclusion.

For the WST, 74 studies fulfilled the inclusion criteria yielding 165 independent samples (13,524 participants). Outlier identification using Cook's distance indicated one leverage point, so the respective study was omitted from all further analyses resulting in 164 samples overall. Included studies covered a time span of 12 years (publication years: 1997–2008). Since studies describing results of 10 samples did not report mean age, they had to be omitted from multiple weighted linear meta-regression. Thus, analysis was based on 71 studies yielding 154 independent samples (12,555 participants; weighted mean age of samples  =  42.18, *SD*  =  17.5; means ranging from 16.6 to 75.6 years). Samples from individual studies were generally small and comprised mainly healthy volunteers (15.8% patients out of 13,524 participants and 16.0% patients out of 12,555 participants). Figure 1 provides a flowchart illustrating the process of study inclusion.

Since results of the two dependent measures (MWT and WST) were reported only incidentally in primary studies (e.g., to describe sample characteristics), there was no publication or selection bias to be expected in extracted data. Only native German-speaking participants were included in both analyses. All studies were in English or German language and performed either in Austria, Germany, or Switzerland. References of included studies can be found in the supporting information (File S1).

## Results

### Analysis 1 (MWT)

**Figure 2:**
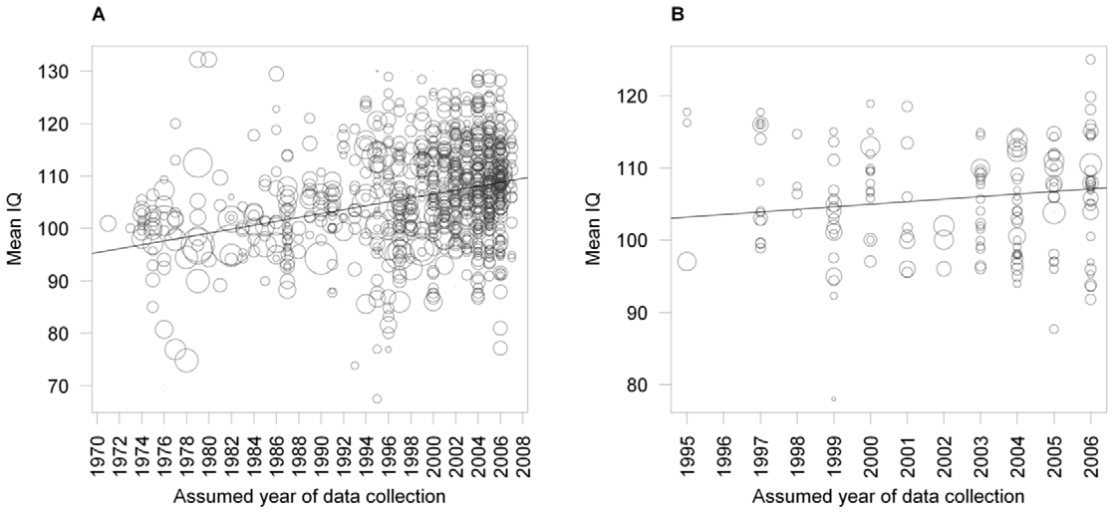
Intelligence test gains on two measures of crystallized intelligence. *Note.* Symbol size is varied according to study weight. (A) Mean IQ of 960 independent samples for the MWT in assumed year of study performance 1971–2007. (B) Mean IQ of 164 samples for the WST in assumed year of study performance 1995–2006.

Analyses of studies employing the MWT as the dependent measure yielded results as follows. First, weighted single meta-regression showed a significant positive slope for assumed year of study performance on mean IQ (Figure 2, panel A). Second, weighted multiple meta-regression yielded a significant positive slope for assumed year of study performance, but no significant effect of age (Table 1). Third, delta IQ showed an average gain of 2.29 IQ points per decade. Fourth, no effect of assumed year of study performance on within-study variances was found (Table 2).

**Table 1:** Single and Multiple Weighted Regression Analyses for MWT and WST Scores.

	*k*	*b*	*SE*	*p*	*R* ^2^
MWT (1971–2007)					
Single regression	960				.14
Intercept		95.74	0.81	<.001	
Assumed year of study performance		0.37	0.03	<.001	
Multiple regression	900				.14
Intercept		96.56	1.35	<.001	
Assumed year of study performance		0.37	0.03	<.001	
Mean age		−0.02	0.02	.448	
WST (1995–2006)					
Single regression	164				.03
Intercept		103.17	1.43	<.001	
Assumed year of study performance		0.36	0.17	.042	
Multiple regression	154				.06
Intercept		100.29	1.89	<.001	
Assumed year of study performance		0.20	0.18	.265	
Mean age		0.09	0.03	.007	

*Note.* All measures in regression analyses were weighted according to sample size; slopes of assumed year of study performance can be interpreted as average gain per year in IQ points; *k* denotes number of included samples; *b* denotes the unstandardized regression coefficients; *SE*  =  standard error; *R* ^2^ denotes percentage of variance of data that is explained by the model.

**Table 2:** Weighted Regression of Assumed Year of Study Performance on Within-Study Variances.

	*k*	*b*	*SE*	*p*	*R* ^2^
Regression (MWT; 1971-2007)	795				<.01
Intercept		0.22	0.10	.03	
Assumed year of study performance		−0.01	<0.01	.07	
Regression (WST; 1995–2006)	146				<.01
Intercept		−0.13	0.21	.55	
Assumed year of study performance		0.02	0.03	.50	

*Note*. All measures in regression analyses were weighted according to sample size; only studies reporting standard deviations or variances of samples' mean IQ were included; *k* denotes number of included samples; *b* denotes the unstandardized regression coefficients; *SE*  =  standard error; *R* ^2^ denotes percentage of variance of data that is explained by the model.

Finally, a weighted multiple stepwise regression was calculated. Variance inflation factors (VIFs) indicated high collinearity of predictors (VIFs > 10). Therefore, following the procedure as proposed by Cohen and colleagues [27], in each turn, covariates showing the highest VIFs were dropped until no VIF displayed a value > 10 (covariates were dropped in the following order: assumed year of study performance * publication status, publication status, assumed year of study performance * publication language, publication language, assumed year of study performance * sample type, sample type), resulting in the best fitting (and most parsimonious) regression model with assumed year of study performance as the only meaningful predictor for mean intelligence test performance.

### Analysis 2 (WST)

First, weighted single meta-regression yielded a significant positive slope for assumed year of study performance (Figure 2, panel B). Second, weighted multiple meta-regression did not reach significance for assumed year of study performance, but showed a significant influence of age (Table 1). Third, delta IQ showed an average gain of 0.63 IQ points per decade. Fourth, no effect of assumed year of study performance on within-study variances was found (Table 2).

## Discussion

Our results clearly demonstrate that generational IQ gains on crystallized intelligence tasks in the general population are in progress in German-speaking countries. Moreover, our observed evidence suggests that these IQ gains are of comparable size as fluid IQ gains and exhibit virtually the same typical linear increase as reported for fluid intelligence [9]. These results are consistent with previous findings of Flynn [11] and Voracek [8] and present several points of interest.

First, even though there is evidence for stagnation and even a reversal of the Flynn effect in Scandinavian countries [2], [9], IQ gains apparently are still ongoing in German-speaking countries. Both meta-regressions yielded significant positive slopes for mean IQ regarding assumed year of study performance, indicating test score gains of about 3.5 points per decade. Although these slopes were based on two different test measures and completely independent samples as well as different time spans, they exhibited a striking similarity of strength. These virtually identical slopes for different time spans indicate that gains have been continuing rather consistently and are not largely due to an increase prior 1995.

Second, in multiple meta-regressions, participants mean age showed no significant influence on mean IQ, but the positive slope for year of study performance remained stable for the MWT. In the WST, age reached significance but publication year of studies failed to do so, although coefficients still pointed in the expected direction. This may be explained by the fact that the WST is a relatively novel measure and dissemination of test measures takes its time. Therefore, most studies included in this analysis were published over a relatively short time span and intelligence test gains might have been masked by higher performance of older participants as suggested by our data (positive slope for age) and as is to be expected on measures of crystallized intelligence [28]. However, due to sample characteristics in the present study, it could not be ruled out that differences in respect to gains exist between subjects younger than age 17 and subjects that are 17 years of age or older, as has been demonstrated in a previous study [12]. Indeed, considering this evidence of Flynn [12], a threat of possible underestimation of the gains in the present study due to specific characteristics of participants' age seems unlikely since only a negligible portion of samples had a smaller mean age than 17.Notably, the upwards shift of intelligence test scores affected the whole ability distribution and not just parts of it. Decreasing population variance in IQ test performance as reported by Colom and colleagues [29] in a Spanish sample could be ruled out as cause for the IQ gains in the present study, since a regression on weighted within-study variances over time produced no evidence for diminishing variances.

Third, in contrast to the majority of findings from Anglo-American countries, in German-speaking countries the Flynn effect is observable for the crystallized domain of intelligence. The average gain on the MWT amounted to 3.7 IQ points per decade and was virtually identical for the WST. It should be noted that Jensen's delta IQs were somewhat smaller than the slopes of regression analyses, thus indicating a slightly smaller gain, although signs indicated the same direction of changes. However, delta IQs are only fitted on a portion of available data, whereas regression models are based on all provided data points. Therefore, results of delta IQ calculations should be considered as an additional indicator of the presence of test score gains, but numerical values are to be interpreted with caution.

Fourth, the Flynn effect is usually assessed in samples displaying very specific characteristics (usually large, healthy, population-based, male samples). In the present study, the effect was ascertainable through meta-analytic aggregation of many small, non-representative, and healthy as well as patient-based samples. Assumed year of study performance turned out to be the optimum available predictor even when accounting for publication language (English vs German), publication status (published vs unpublished), and sample type (patients vs normals). This finding indicates a remarkable robustness of the Flynn effect in our sample.

So the accumulated evidence speaks a very clear language regarding the presence of these gains, but their cause still needs to be investigated. Our results emphasize the importance of test renorming even for measures of crystallized intelligence, at least in German-speaking populations. In the end, the present findings accentuate that crystallized intelligence should not be disregarded with respect to research on the Flynn effect. Since evidently average intelligence test scores of the general population fluctuate, stability of crystallized intelligence in different populations should not be taken for granted.

In conclusion, our results show that the Flynn effect still progresses and can be accounted for measures of crystallized intelligence in German-speaking countries. These IQ gains are robust enough to be demonstrated in a great many of small and non-representative samples, thus indicating pervasiveness of this still poorly understood phenomenon.

## Supporting Information

File S1 References of Included Studies
(0.19 MB DOC)
