# Supplementary material for: Pervasiveness of the IQ Rise: A Cross-Temporal Meta-Analysis
Source: PLoS One. 2010 Dec 22;5(12):e14406. doi: 10.1371/journal.pone.0014406 (PMC3008673; doi:10.1371/journal.pone.0014406)
Supplement: File S1 — References of Included Studies (0.19 MB DOC) [file pone.0014406.s001.doc]

**References of included studies**

1. Adler C (1988) Psychodiagnostische Untersuchung von Patienten mit Verdacht mit hirnorganisch bedingtes Leistungsversagen unter besonderer Berücksichtigung des Lerntestkonzeptes [Psychodiagnostic examinations of patients suspected of hirnorganical induced failure of schievement considering learn test concepts] [doctoral dissertation]. Leipzig: University of Leipzig.

2. Aleksic M, Huff W, Hoppmann B, Heckenkamp J, Pukrop R, et al. (2006) Cognitive function remains unchanged after endarterectomy of unilateral internal carotid artery stenosis under local anaesthesia. European Journal of Vascular and Endovascular Surgery 31: 616-621.

3. Altgassen M, Kliegel M, Rendell P, Henry JD, Zolling J (2008) Prospective memory in schizophrenia: The impact of varying retrospective-memory load. Journal of Clinical and Experimental Neuropsychology 30: 777-788.

4. Altgassen M, Phillips L, Kopp U, Kliegel M (2007) Role of working memory components in planning performance of individuals with Parkinson's disease. Neuropsychologia 45: 2393-2397.

5. Altgassen M, Zollig J, Kopp U, Mackinlay R, Kliegel M (2007) Patients with Parkinson's disease can successfully remember to execute delayed intentions. Journal of the International Neuropsychological Society 13: 888-892.

6. Anderlan MJA (1984) Eine Untersuchung zum differentialdiagnostischen Wert subjektiver und objektiver Leistungsbeeinträchtigungen bei Schizophrenen im Vergleich zu Hirnorganikern und Konversionsneurotikern [An investigation to the differential diagnostic worth of subjective and objective achievement impairments in schizophrene subjects in comparison to subjects suffering from brainorganic psychosyndrom and subjects suffering from transference neurosis] [doctoral dissertation]. Innsbruck: University of Innsbruck.

7. Assenbaum H (1978) Der Einfluß von Befindensstörungen auf Leistungs- und Intelligenztestergebnisse bei jungen Erwachsenen [Influences of affectivity disorders on achievement and intelligence test results in young adults] [doctoral thesis]. Nürnberg: University of Erlangen-Nürnberg.

8. Bark R, Dieckmann S, Bogerts B, Northoff G (2005) Deficit in decision making in catatonic schizophrenia: An exploratory study. Psychiatry Research 134: 131-141.

9. Barnett W, Richter P, Mundt C (1999) Empirische Studie zum Konstrukt primärer und sekundärer schizophrener Negativsymptomatik [An empirical study of the construct of primary and secundary schizophrenic negative symptomatic]. In: Bräunig P, editor. Motorische Störungen bei schizophrenen Psychosen [Motor disfunctions in schizophrenic psychoses]. Stuttgart: Schattauer. pp. 179-187.

10. Bartha L, Benke T, Bauer G, Trinka E (2005) Interictal language functions in temporal lobe epilepsy. Journal of Neurology Neurosurgery and Psychiatry 76: 808-814.

11. Bartha L, Marksteiner J, Bauer G, Benke T (2002) Persistent cognitive deficits associated with lithium intoxication: A neuropsychological case description. Cortex 38: 743-752.

12. Beinhoff U, Tumani H, Brettschneider J, Bittner D, Riepe MW (2008) Gender-specificities in Alzheimer's disease and mild cognitive impairment. Journal of Neurology 255: 117-122.

13. Bellebaum C, Schafers L, Schoch B, Wanke I, Stolke D, et al. (2004) Clipping versus coiling: Neuropsychological follow up after aneurysmal subarachnoid haemorrhage (SAH). Journal of Clinical and Experimental Neuropsychology 26: 1081-1092.

14. Bender W, Vaitl P, Schnattinger H (1985) Cognitive dysfunctions in schizophrenic patients - a controlled study. European Archives of Psychiatry and Clinical Neuroscience 235: 97-101.

15. Bengner T, Fortmeier C, Malina T, Lindenau M, Voges B, et al. (2006) Sex differences in face recognition memory in patients with temporal lobe epilepsy, patients with generalized epilepsy, and healthy controls. Epilepsy & Behavior 9: 593-600.

16. Bengner T, Haettig H, Merschhemke M, Dehnicke C, Meencke HJ (2003) Memory assessment during the intracarotid amobarbital procedure - Influence of injection order. Neurology 61: 1582-1587.

17. Bengner T, Malina T (2007) Dissociation of short- and long-term face memory: Evidence from long-term recency effects in temporal lobe epilepsy. Brain and Cognition 64: 189-200.

18. Bengner T, Malina T, Lindenau M, Voges B, Goebell E, et al. (2006) Face memory in MRI-positive and MRI-negative temporal lobe epilepsy. Epilepsia 47: 1904-1914.

19. Benke T, Delazer M, Bartha L, Auer A (2003) Basal ganglia lesions and the theory of fronto-subcortical loops: Neuropsychological findings in two patients with left caudate lesions. Neurocase 9: 70-85.

20. Benke T, Hohenstein C, Poewe W, Butterworth B (2000) Repetitive speech phenomena in Parkinson's disease. Journal of Neurology Neurosurgery and Psychiatry 69: 319-325.

21. Benke T, Kurzthaler I, Schmidauer C, Moncayo R, Donnemiller E (2002) Mania caused by a diencephalic lesion. Neuropsychologia 40: 245-252.

22. Benos J (1978) Langzeitbeobachtung und psychopathometrische Untersuchung eines Falles von imitierter Gemeinschaftspsychose [Long-term observation and psychopathometric investigation of a case of imitated community psychosis]. Psycho 4: 699-701.

23. Benos J (1980) Changes of signs and delusional behavior in aged chronic schizophrenics. Fortschritte der Neurologie Psychiatrie 48: 513-555.

24. Benos J (1984) Psychopathometric cross-sectional diagnosis in aged chronic schizophrenics. Fortschritte der Neurologie Psychiatrie 52: 223-236.

25. Bittner D, Gron G, Schirrmeister H, Reske SN, Riepe MW (2005) [F-18]FDG-PET in patients with Alzheimer's disease: Marker of disease spread. Dementia and Geriatric Cognitive Disorders 19: 24-30.

26. Blaha L, Pater W (1979) Stability and reliability of a brief intelligence test (MWT-B) to long-stay psychiatric patients. Nervenarzt 50: 196-198.

27. Blöink M, Sabarowski F (1986) Die Herzschrittmacherimplantation: Eine Ursache der Depression im Alter? [Implantation of pacemakers: A cause of depression in elder subjects?]. In: Bergener M, Kranzhoff EUM, Husser JM, editors. Depressionen im Alter [Depressions in old age]. Darmstadt: Steinkopff.

28. Bockelmann I, Darius S, McGauran N, Robra BP, Peter B, et al. (2002) The psychological effects of exposure to mixed organic solvents on car painters. Disability and Rehabilitation 24: 455-461.

29. Bockelmann I, Pfister EA, Peters B, Duchstein S (2004) Psychological effects of occupational exposure to organic solvent mixtures on printers. Disability and Rehabilitation 26: 798-807.

30. Boeker H, Kleiser M, Lehman D, Jaenke L, Bogerts B, et al. (2006) Executive dysfunction, self, and ego pathology in schizophrenia: an exploratory study of neuropsychology and personality. Comprehensive Psychiatry 47: 7-19.

31. Bolte S, Holtmann M, Poustka F, Scheurich A, Schmidt L (2007) Gestalt perception and local-global processing in high-functioning autism. Journal of Autism and Developmental Disorders 37: 1493-1504.

32. Bonatti E, Zamarian L, Wagner M, Benke T, Hollosi P, et al. (2008) Making decisions and advising decisions in traumatic brain injury. Cognitive and Behavioral Neurology 21: 164-175.

33. Borgwardt SJ, McGuire PK, Aston J, Gschwandtner U, Pfluger MO, et al. (2008) Reductions in frontal, temporal and parietal volume associated with the onset of psychosis. Schizophrenia Research 106: 108-114.

34. Borgwardt SJ, Riecher-Rossler A, Dazzan P, Chitnis X, Aston J, et al. (2007) Regional gray matter volume abnormalities in the at risk mental state. Biological Psychiatry 61: 1148-1156.

35. Bornschein S, Hausteiner C, Pohl C, Jahn T, Angerer J, et al. (2008) Pest controllers: A high-risk group for multiple chemical sensitivity (MCS)? Clinical Toxicology 46: 193-200.

36. Borsutzky S, Fujiwara E, Brand M, Markowitsch HJ (2008) Confabulations in alcoholic Korsakoff patients. Neuropsychologia 46: 3133-3143.

37. Bosbach S, Kornblum C, Schroder R, Wagner M (2003) Executive and visuospatial deficits in patients with chronic progressive external ophthalmoplegia and Kearns-Sayre syndrome. Brain 126: 1231-1240.

38. Bosshardt HG (2002) Effects of concurrent cognitive processing on the fluency of word repetition: comparison between persons who do and do not stutter. Journal of Fluency Disorders 27: 93-114.

39. Bosshardt HG, Ballmer W, de Nil LF (2002) Effects of category and rhyme decisions on sentence production. Journal of Speech Language and Hearing Research 45: 844-857.

40. Bosshardt HG, Fransen H (1996) Online sentence processing in adults who stutter and adults who do not stutter. Journal of Speech and Hearing Research 39: 785-797.

41. Bosshardt HG, Sappok C, Knipschild M, Holscher C (1997) Spontaneous imitation of fundamental frequency and speech rate by nonstutterers and stutterers. Journal of Psycholinguistic Research 26: 425-448.

42. Bottlender R, Buchberger A, Hoff P, Moller HJ (1999) Decision-making and delusion. A study on decision-making in deluded, depressive and healthy subjects. Nervenarzt 70: 987-992.

43. Brand A, Kopmann S, Herzog M (2004) Intact feature fusion in schizophrenic patients. European Archives of Psychiatry and Clinical Neuroscience 254: 281-288.

44. Brand A, Kopmann S, Marbach S, Heinze M, Herzog MH (2005) Intact and deficient feature fusion in schizophrenia. European Archives of Psychiatry and Clinical Neuroscience 255: 413-418.

45. Brand M, Fujiwara E, Borsutzky S, Kalbe E, Kessler J, et al. (2005) Decision-making deficits of Korsakoff patients in a new gambling task with explicit rules: Associations with executive functions. Neuropsychology 19: 267-277.

46. Brand M, Pawlikowski M, Labudda K, Laier C, von Rothkirch N, et al. (2009) Do amnesic patients with Korsakoff's syndrome use feedback when making decisions under risky conditions? An experimental investigation with the Game of Dice Task with and without feedback. Brain and Cognition 69: 279-290.

47. Braun A, Muller UA, Muller R, Leppert K, Schiel R (2004) Structured treatment and teaching of patients with Type 2 diabetes mellitus and impaired cognitive function - the DICOF trial. Diabetic Medicine 21: 999-1006.

48. Braun M, Finke C, Ostendorf F, Lehmann TN, Hoffmann KT, et al. (2008) Reorganization of associative memory in humans with long-standing hippocampal damage. Brain 131: 2742-2750.

49. Brockhaus C (1998) Stimmung und Problemlösen in der Depression [Mood and problem solving in depressions]. Göttingen: Cuvillier.

50. Brockhaus-Dumke A, Tendolkar I, Pukrop R, Schultze-Lutter F, Klosterkotter J, et al. (2005) Impaired mismatch negativity generation in prodromal subjects and patients with schizophrenia. Schizophrenia Research 73: 297-310.

51. Brokate B, Bernsdorff K, Braamhorst W, Eling P, Hildebrandt H (2008) Object alternation in alcohol dependent patients without amnesic syndrome. Zeitschrift fur Neuropsychologie 19: 33-40.

52. Brokate B, Hildebrandt H, Eling P, Fichtner H, Runge K, et al. (2003) Frontal lobe dysfunctions in Korsakoff's syndrome and chronic alcoholism: Continuity or discontinuity? Neuropsychology 17: 420-428.

53. Brune M, Lissek S, Fuchs N, Witthaus H, Peters S, et al. (2008) An fMRI study of theory of mind in schizophrenic patients with "passivity" symptoms. Neuropsychologia 46: 1992-2001.

54. Bublak P, Finke K, Krummenacher J, Preger R, KyllingsbaeK S, et al. (2005) Usability of a theory of visual attention (TVA) for parameter-based measurement of attention II: Evidence from two patients with frontal or parietal damage. Journal of the International Neuropsychological Society 11: 843-854.

55. Bublak P, Muller U, Gron G, Reuter M, von Cramon DY (2002) Manipulation of working memory information is impaired in Parkinson's disease and related to working memory capacity. Neuropsychology 16: 577-590.

56. Buchmann A, Mondadori CRA, Haenggi J, Aerni A, Vrticka P, et al. (2008) Prion protein M129V polymorphism affects retrieval-related brain activity. Neuropsychologia 46: 2389-2402.

57. Buchta M, Kiesswetter E, Otto A, Schaller KH, Seeber A, et al. (2003) Longitudinal study examining the neurotoxicity of occupational exposure to aluminium-containing welding fumes. International Archives of Occupational and Environmental Health 76: 539-548.

58. Buchta M, Kiesswetter E, Schaper M, Zschiesche W, Schaller KH, et al. (2005) Neurotoxicity of exposures to aluminium welding fumes in the truck trailer construction industry. Environmental Toxicology and Pharmacology 19: 677-685.

59. Bühler K-E, Gross M, Jurgensen R (1991) Psychometric differentiation of schizophrenic and schizoaffective psychoses. Schweizer Archiv für Neurologie und Psychiatrie 142: 535-552.

60. Bühler K-E, Vogt H (1987) Cognitive performance profile in schizophrenic patients. Schweizer Archiv für Neurologie und Psychiatrie 138: 25-44.

61. Bussmann C, Sauer M, Kessler J, Hautzinger M, Markowitsch HJ (1988) Wiedererkennensleistung und Funktionsrestitution bei abklingenden depressiven Erkrankungen: Eine Studie mit visueller unilateraler Exposition affektiv getönten Reizmaterials [Recognition performance and functional restitution in subsiding depressive disorders: A unilateral exposition study employing affective stimulus material]. Zeitschrift für Klinische Psychologie 17: 307-318.

62. Ciesielski AS, Samson S, Steinhoff BJ (2006) Neuropsychological and psychiatric impact of add-on titration of pregabalin versus levetiracetam: A comparative short-term study. Epilepsy & Behavior 9: 424-431.

63. Colla M, Ende G, Alm B, Deuschle M, Heuser I, et al. (2008) Cognitive MR spectroscopy of anterior cingulate cortex in ADHD: Elevated choline signal correlates with slowed hit reaction times. Journal of Psychiatric Research 42: 587-595.

64. Csomor PA, Stadler RR, Feldon J, Yee BK, Geyer MA, et al. (2008) Haloperidol differentially modulates prepulse inhibition and P50 suppression in healthy humans stratified for low and high gating levels. Neuropsychopharmacology 33: 497-512.

65. Dannlowski U, Kersting A, Arolt V, Lalee-Mentzel J, Donges US, et al. (2006) Unimpaired automatic processing of verbal information in the course of clinical depression. Depression and Anxiety 23: 325-330.

66. Dannlowski U, Kersting A, Donges US, Lalee-Mentzel J, Arolt V, et al. (2006) Masked facial affect priming is associated with therapy response in clinical depression. European Archives of Psychiatry and Clinical Neuroscience 256: 215-221.

67. Dannlowski U, Ohrmann P, Konrad C, Domschke K, Bauer J, et al. (2009) Reduced amygdala-prefrontal coupling in major depression: association with MAOA genotype and illness severity. International Journal of Neuropsychopharmacology 12: 11-22.

68. Daun H, Hilfinger E, Jakob H, Lehrl S (1978) Sprichworterklären und Bildergeschichtendeuten bei Psychosen [Explanation of proverbs and interpretation of comic strips in psychoses]. Medizinische Welt 29: 1263-1267.

69. Delazer M, Girelli L, Benke T (1999) Arithmetic reasoning and implicit memory: A neuropsychological study on amnesia. Cortex 35: 615-627.

70. Delazer M, Karner E, Zamarian L, Donnemiller E, Benke T (2006) Number processing in posterior cortical atrophy - A neuropsycholgical case study. Neuropsychologia 44: 36-51.

71. Delazer M, Lochy A, Jenner C, Domahs F, Benke T (2002) When writing 0 (zero) is easier than writing O (o): A neuropsychological case study of agraphia. Neuropsychologia 40: 2167-2177.

72. Diehl A, Croissant B, Batra A, Mundle G, Nakovics H, et al. (2007) Alcoholism in women: Is it different in onset and outcome compared to men? European Archives of Psychiatry and Clinical Neuroscience 257: 344-351.

73. Dietl T, Urbach H, Helmstaedter C, Staedtgen M, Szentkuti A, et al. (2004) Persistent severe amnesia due to seizure recurrence after unilateral temporal lobectomy. Epilepsy & Behavior 5: 394-400.

74. Dobel C, Cohen R, Berg P, Rockstroh B, Koebbel P, et al. (1999) Event-related potential correlates of verbal and pictorial feature comparison in aphasics and controls. Psychophysiology 36: 47.

75. Dobmann-Murrmann G (1981) Der Einfluß von Angst und Befindensstörungen auf Leistungs- und Intelligenztestergebnisse bei psychiatrisch unauffälligen Medizinstudenten [Influences of anxiety and mood disorders on performance and intelligence test scores in psychiatric unconspicuous medical students] [doctoral thesis]. Nürnberg: University of Erlangen-Nürnberg.

76. Dohrenbusch R, Scholz OB, Ott R (2006) Conscious and preconscious uses of memory in patients with depressive and somatoform disorders. Journal of Psychopathology and Behavioral Assessment 28: 69-77.

77. Doldi S (1985) Kognitive Störungen bei Anorexia nervosa [Cognitive disorders in Anorexia nervosa] [doctoral thesis]. Innsbruck: University of Innsbruck.

78. Donges US, Kersting A, Dannlowski U, Lalee-Mentzel J, Arolt V, et al. (2005) Reduced awareness of others' emotions in unipolar depressed patients. Journal of Nervous and Mental Disease 193: 331-337.

79. Dyck M, Winbeck M, Leiberg S, Chen Y, Gur RC, et al. (2008) Recognition profile of emotions in natural and virtual faces. PLoS ONE 3: e3682.

80. Ebner F, Tepest R, Dani I, Pfeiffer U, Schulze TG, et al. (2008) The hippocampus in families with schizophrenia in relation to obstetric complications. Schizophrenia Research 104: 71-78.

81. Ebner NC, Freund AM, Baltes PB (2006) Developmental changes in personal goal orientation from young to late adulthood: From striving for gains to maintenance and prevention of losses. Psychology and Aging 21: 664-678.

82. Eder-Sommer G, Romero B (1996) Verlangsamung des mentalen Durchmusterns (memory scanning) bei älteren Depressiven: Eine kontrollierte klinische Untersuchung [Deceleration of mental memory scanning in elderly depressive subjects]. Zeitschrift für Neuropsychologie 7: 48-60.

83. Ehrenreich H, Fischer B, Norra C, Schellenberger F, Stender N, et al. (2007) Exploring recombinant human erythropoietin in chronic progressive multiple sclerosis. Brain 130: 2577-2588.

84. Ehrenreich H, Hinze-Selch D, Stawicki S, Aust C, Knolle-Veentjer S, et al. (2007) Improvement of cognitive functions in chronic schizophrenic patients by recombinant human erythropoietin. Molecular Psychiatry 12: 206-220.

85. Ehrensperger MM, Grether A, Romer G, Berres M, Monsch AU, et al. (2008) Neuropsychological dysfunction, depression, physical disability, and coping processes in families with a parent affected by multiple sclerosis. Multiple Sclerosis 14: 1106-1112.

86. El Sharkawy J, Groth K, Vetter C, Beraldi A, Fast K (2008) False memories of emotional and neutral words. Behavioural Neurology 19: 7-11.

87. Endrass T, Klawohn J, Schuster F, Kathmann N (2008) Overactive performance monitoring in obsessive-compulsive disorder: ERP evidence from correct and erroneous reactions. Neuropsychologia 46: 1877-1887.

88. Engel K, Ahrens HJ (1979) Interaction diagnostics - empirical comparison of Bales Scale, expert rating, Giessentest, Joint Rorschach Test, and Simfam. Gruppenpsychotherapie und Gruppendynamik 14: 374-392.

89. Eppinger B, Kray J, Mock B, Mecklinger A (2008) Better or worse than expected? Aging, learning, and the ERN. Neuropsychologia 46: 521-539.

90. Eschen A, Freeman J, Dietrich T, Martin M, Ellis J, et al. (2007) Motor brain regions are involved in the encoding of delayed intentions: A fMRI study. International Journal of Psychophysiology 64: 259-268.

91. Eschweiler GW, Vonthein R, Bode R, Huell M, Conca A, et al. (2007) Clinical efficacy and cognitive side effects of bifrontal versus right unilateral electroconvulsive therapy (ECT): A short-term randomised controlled trial in pharmaco-resistant major depression. Journal of Affective Disorders 101: 149-157.

92. Fajnor K (1986) Zur prämorbiden Persönlichkeit monopolar endogen Depressiver: Zusammenhänge zwischen Persönlichkeit und Symptomatik [Premorbid personality of monopolar endogenous depressive subjects: Correlations of personality and symptoms] [doctoral dissertation]. Innsbruck: University of Innsbruck.

93. Falkai P, Honer WG, Kamer T, Dustert S, Vogeley K, et al. (2007) Disturbed frontal gyrification within families affected with schizophrenia. Journal of Psychiatric Research 41: 805-813.

94. Falkai P, Schneider-Axmann T, Honer WG, Vogeley K, Schonell H, et al. (2003) Influence of genetic loading, obstetric complications and premorbid adjustment on brain morphology in schizophrenia: A MRI study. European Archives of Psychiatry and Clinical Neuroscience 253: 92-99.

95. Fauser S, Talazko J, Wagner K, Ziyeh S, Jarius S, et al. (2005) FDG-PET and MRI in potassium channel antibody-associated non-paraneoplastic limbic encephalitis: correlation with clinical course and neuropsychology. Acta Neurologica Scandinavica 111: 338-343.

96. Ferstl EC, Walther K, Guthke T, von Cramon DY (2005) Assessment of story comprehension deficits after brain damage. Journal of Clinical and Experimental Neuropsychology 27: 367-384.

97. Finke C, Braun M, Ostendorf F, Lehmann TN, Hoffmann KT, et al. (2008) The human hippocampal formation mediates short-term memory of colour-location associations. Neuropsychologia 46: 614-623.

98. Finke K, Bublak P, Krummenacher J, Kyllingsbaek S, Muller HJ, et al. (2005) Usability of a theory of visual attention (TVA) for parameter-based measurement of attention I: Evidence from normal subjects. Journal of the International Neuropsychological Society 11: 832-842.

99. Fischer B, Fischer U (1983) Nutzung der reduzierten subjektiven Information bei der Intelligenzmessung durch Kleincomputer [Utilization of reducted subjective information in intelligence assessment through miniature computers]. Grundlagenstudien aus Kybernetik und Geisteswissenschaft 24: 123-128.

100. Fischer B, Lehrl S, Weber E, Gundertremy U, Fischer U (1981) Cerebrovascular insufficiency and drug-compliance. Zeitschrift fur Gerontologie 14: 145-152.

101. Fischer I, Merten T (2001) Eine Untersuchung zur Methodik der Kreativitätsdiagnostik [An investigation of methods of diagnostics of creativity]. Zeitschrift für Differentielle und Diagnostische Psychologie 22: 305-316.

102. Fischhof PK, Friedmann A, Moslinger-Gehmayr R (2001) The influence of acquired intelligence on cerebral impairment in the elderly. Drugs of Today 37: 697-702.

103. Forstmeier S, Maercker A (2008) Motivational reserve: Lifetime motivational abilities contribute to cognitive and emotional health in old age. Psychology and Aging 23: 886-899.

104. Freudenmann RW, Spitzer M (2001) Computer assisted questioning as a basis for a modern quality assurance in psychiatry: Results from pilot studies. Nervenarzt 72: 40-51.

105. Fritz N, Glogau S, Hoffmann J, Rademacher M, Elger CE, et al. (2005) Efficacy and cognitive side effects of tiagabine and topiramate in patients with epilepsy. Epilepsy & Behavior 6: 373-381.

106. Fujiwara E, Brand M, Borsutzky S, Steingass HP, Markowitsch HJ (2008) Cognitive performance of detoxified alcoholic Korsakoff syndrome patients remains stable over two years. Journal of Clinical and Experimental Neuropsychology 30: 576-587.

107. Fujiwara E, Brand M, Kracht L, Kessler J, Diebel A, et al. (2008) Functional retrograde amnesia: A multiple case study. Cortex 44: 29-45.

108. Geretsegger C, Nickel M, Judendorfer B, Rochowanski E, Novak E, et al. (2007) Propofol and methohexital as anesthetic agents for electroconvulsive therapy: A randomized, double-blind comparison of electroconvulsive therapy seizure, quality, therapeutic efficacy, and cognitive performance. Journal of ECT 23: 239-243.

109. Girelli L, Bartha L, Delazer M (2002) Strategic learning in the rehabilitation of semantic knowledge. Neuropsychological Rehabilitation 12: 41-61.

110. Girelli L, Semenza C, Delazer M (2004) Inductive reasoning and implicit memory: evidence from intact and impaired memory systems. Neuropsychologia 42: 926-938.

111. Gleissner U, Elger CE (2001) The hippocampal contribution to verbal fluency in patients with temporal lobe epilepsy. Cortex 37: 55-63.

112. Gleissner U, Helmstaedter C, Elger CE (2002) Memory reorganization in adult brain: observations in three patients with temporal lobe epilepsy. Epilepsy Research 48: 229-234.

113. Gleissner U, Helmstaedter C, Quiske A, Elger CE (1998) The performance-complaint relationship in patients with epilepsy: a matter of daily demands? Epilepsy Research 32: 401-409.

114. Gleissner U, Helmstaedter C, Schramm J, Elger CE (2002) Memory outcome after selective amygdalohippocampectomy: A study in 140 patients with temporal lobe epilepsy. Epilepsia 43: 87-95.

115. Gleissner U, Sassen R, Lendt M, Clusmann H, Elger CE, et al. (2002) Pre- and postoperative verbal memory in pediatric patients with temporal lobe epilepsy. Epilepsy Research 51: 287-296.

116. Goder R, Boigs M, Braun S, Friege L, Fritzer G, et al. (2004) Impairment of visuospatial memory is associated with decreased slow wave sleep in schizophrenia. Journal of Psychiatric Research 38: 591-599.

117. Goder R, Fritzer G, Gottwald B, Lippmann B, Seeck-Hirschner M, et al. (2008) Effects of olanzapine on slow wave sleep, sleep spindles and sleep-related memory consolidation in schizophrenia. Pharmacopsychiatry 41: 92-99.

118. Goder R, Scharffetter F, Aldenhoff JB, Fritzer G (2007) Visual declarative memory is associated with non-rapid eye movement sleep and sleep cycles in patients with chronic non-restorative sleep. Sleep Medicine 8: 503-508.

119. Goeder R, Aldenhoff JB, Boigs M, Braun S, Koch J, et al. (2006) Delta power in sleep in relation to neuropsychological performance in healthy subjects and schizophrenia patients. Journal of Neuropsychiatry and Clinical Neurosciences 18: 529-535.

120. Gohl K-H (1992) Beurteilung von therapeutischen Maßnahmen, auf der Grundlage von Einzelfallanalysen, am Beispiel der geschlossenen forensisch-psychiatrischen Klinik der Landesnervenklinik Neuruppin [Evaluation of therapeutic methods on basis of case-studies examplified in the forensic-psychiatric county hospital in Neuruppin] [doctoral dissertation]. Leipzig: University of Leipzig.

121. Golka K, Kiesswetter E, Kieper H, Blaszkewicz M, Hallier E, et al. (2000) Psychological effects upon exposure to polyhalogenated dibenzodioxins and dibenzofurans. Chemosphere 40: 1271-1275.

122. Goltz L (1982) Schmerzangaben bei Patienten mit psychovegetativen und reaktiven Depressionszuständen im Vergleich zu solchen bei endogener Depression und bei organischer Begründbarkeit [Reports of pain in patients suffering from psychovegetative and reactive depressions compared to patients suffering from endogenous depression and organic bases] [doctoral dissertation]. Nürnberg: University of Erlangen-Nürnberg.

123. Gorlicki C, Schauble R (1997) How valid is the WST for intelligence assessment? Zeitschrift fur Klinische Psychologie Psychiatrie und Psychotherapie 45: 367-375.

124. Gottwald B, Wilde B, Mihajlovic Z, Mehdorn HM (2004) Evidence for distinct cognitive deficits after focal cerebellar lesions. Journal of Neurology Neurosurgery and Psychiatry 75: 1524-1531.

125. Gradauer L (1988) Hirnleistungsstörung und Persönlichkeitsveränderung bei Multiple Sklerose-Kranken [Cortical disfunctions and changes of personality in subjects suffering from multiple sclerosis] [doctoral dissertation]. Vienna: University of Vienna.

126. Grafl R (1986) Persönlichkeitsstruktur und Persönlichkeitsveränderung bei Insassen des Maßnahmenvollzuges und der Strafhaft [Personality structure and personality changes in prison inmates] [doctoral dissertation]. Vienna: University of Vienna.

127. Grassel E (1993) Using psychopathometric tests for diagnosing dementia - Comparison of Mini-Mental State and the test battery MWT/KAI. Zeitschrift fur Gerontologie 26: 268-274.

128. Greiner A (1977) Zum Sprichworterklären in Multiple-Choice-Form: Eine Untersuchung bei Normalpersonen, Varianten und den 3 Psychosegrundformen [Explanation of proverbs in multiple-choice format: An investigation of normals, variants, and three basic forms of psychosis] [doctoral dissertation]. Nürnberg: University of Erlangen-Nürnberg.

129. Greiner R (1976) Vergleich von Schmerzangaben bei zyklothymer Depression und körperlicher Begründbarkeit [Comparability of pain reports in cyclothymic depressions due to organic reasons]. Erlangen: Straube.

130. Griego JA, Kliegel M (2008) Adult age differences in function concept learning. Aging Neuropsychology and Cognition 15: 1-30.

131. Gron G, Bittner D, Schmitz B, Wunderlich AP, Riepe MW (2002) Subjective memory complaints: Objective neural markers in patients with Alzheimer's disease and major depressive disorder. Annals of Neurology 51: 491-498.

132. Gron G, Bittner D, Schmitz B, Wunderlich AP, Tomczak R, et al. (2003) Variability in memory performance in aged healthy individuals: an fMRI study. Neurobiology of Aging 24: 453-462.

133. Gron G, Brandenburg I, Wunderlich AP, Riepe MW (2006) Inhibition of hippocampal function in mild cognitive impairment: targeting the cholinergic hypothesis. Neurobiology of Aging 27: 78-87.

134. Gron G, Kirstein M, Thielscher A, Riepe MW, Spitzer M (2005) Cholinergic enhancement of episodic memory in healthy young adults. Psychopharmacology 182: 170-179.

135. Gruhn D, Scheibe S (2008) Age-related differences in valence and arousal ratings of pictures from the International Affective Picture System (IAPS): Do ratings become more extreme with age? Behavior Research Methods 40: 512-521.

136. Gruhn D, Scheibe S, Baltes PB (2007) Reduced negativity effect in older adults' memory for emotional pictures: The heterogeneity-homogeneity list paradigm. Psychology and Aging 22: 644-649.

137. Gruhn D, Smith J, Baltes PB (2005) No aging bias favoring memory for positive material: Evidence from a heterogeneity-homogeneity list paradigm using emotionally toned words. Psychology and Aging 20: 579-588.

138. Grünberger J (1977) Psychodiagnostik des Alkoholkranken: Ein methodischer Beitrag zur Bestimmung der Organizität in der Psychiatrie [Psychodiagnostics of alcoholics: A methodological contribution of organicity in psychiatry]. Vienna: Maudrich.

139. Gschwandtner U, Pfluger M, Aston J, Borgwardt S, Drewe M, et al. (2006) Fine motor function and neuropsychological deficits in individuals at risk for schizophrenia. European Archives of Psychiatry and Clinical Neuroscience 256: 201-206.

140. Gudernatsch V (1978) Der Einfluß von Befindensstörungen auf Leistungs- und Intelligenztestergebnisse bei psychiatrisch unauffälligen Patienten [Influences of mood disorders on performance and intelligence test tasks in psychiatric unconspicuous patients] [doctoral thesis]. Nürnberg: University of Erlangen-Nürnberg.

141. Guthke J, Adler C (1990) Empirische Untersuchungsergebnisse zum „dynamischen Testen“ bei der Psychodiagnostik von Hirnorganikern [Results of an empirical investigation on dynamic testing in psychodiagnostics of subjects suffering from brainorganic psychosyndrome]. Zeitschrift für Gerontopsychologie und -psychiatrie 3: 1-12.

142. Habel U, Koch K, Pauly K, Kellermann T, Reske M, et al. (2007) The influence of olfactory-induced negative emotion on verbal working memory: Individual differences in neurobehavioral findings. Brain Research 1152: 158-170.

143. Hach B, Lehrl S, Niedermeier W (1978) Psychopathologische und psychopathometrische Befunde bei Patienten mit Prothesenunverträglichkeits¬erscheinungen [Psychopathological and psychopathometrical results in patients with incompatibility of prostheses]. Deutsche zahnärztliche Zeitschrift 33: 238-244.

144. Haenschel C, Bittner RA, Haertling F, Rotarska-Jagiela A, Maurer K, et al. (2007) Contribution of impaired early-stage visual processing to working memory dysfunction in adolescents with schizophrenia - A study with event-related Potentials and functional magnetic resonance Imaging. Archives of General Psychiatry 64: 1229-1240.

145. Halbig TD, Kopp UA, Wodarz F, Borod JC, Gracies JM, et al. (2008) Dopaminergic modulation of emotional memory in Parkinson's disease. Journal of Neural Transmission 115: 1159-1163.

146. Hamster W, Diener HC (1984) Neuropsychological changes associated with stenoses or occlusions of the carotid arteries - A comparative psychometric study. European Archives of Psychiatry and Clinical Neuroscience 234: 69-73.

147. Heidrich A, Strik WK (1997) Auditory P300 topography and neuropsychological test performance: Evidence for left hemispheric dysfunction in schizophrenia. Biological Psychiatry 41: 327-335.

148. Heinz T (2005) Bedeutung der transkraniellen Dopplersonographie und der diffusionsgewichteten Magnetresonanztomographie zum Nachweis von Mikroembolien und deren neuropsychologische Auswirkungen im Rahmen der Carotisthrombendarteriektomie [Relevance of the transcranial Dopplersonography and the diffusion-weighted magnetic resonance imaging for detection of micro embolisms and resulting neuropsychological effects in the course of carotisthrombendorteriectomy] [doctoral dissertation]. München: Technical University of Munich.

149. Heinze S, Sartory G, Muller BW, de Greiff A, Forsting M, et al. (2006) Neural activation during successful and unsuccessful verbal learning in schizophrenia. Schizophrenia Research 83: 121-130.

150. Helmstaedter C, Elger CE (1992) Beziehungen zwischen Verbalgedächtnis und Sprachleistungen am Beispiel fokaler Temporallappenepilepsien [Relations of verbal memory and lingual performance examplified on subjects suffering from temporal lobe epilepsia]. Zeitschrift für Geriatrie 5: 73-77.

151. Helmstaedter C, Hartmann A, Niese C, Brackmann HH, Sass R (1992) Stage independent and individual courses of neurocognitive deficits in HIV - A follow-up-study of 62 HIV-positive hemophiliacs. Nervenarzt 63: 88-94.

152. Helmstaedter C, Hauff M, Elger CE (1998) Ecological validity of list-learning tests and self-reported memory in healthy individuals and those with temporal lobe epilepsy. Journal of Clinical and Experimental Neuropsychology 20: 365-375.

153. Helmstaedter C, Kemper B, Elger CE (1996) Neuropsychological aspects of frontal lobe epilepsy. Neuropsychologia 34: 399-406.

154. Helmstaedter C, Kurthen M, Lux S, Johanson K, Quiske A, et al. (2000) Long-term clinical, neuropsychological, and psychosocial follow-up in surgically and nonsurgically treated patients with drug-resistant temporal lobe epilepsy. Nervenarzt 71: 629-642.

155. Helmstaedter C, Kurthen M, Lux S, Reuber M, Elger CE (2003) Chronic epilepsy and cognition: A longitudinal study in temporal lobe epilepsy. Annals of Neurology 54: 425-432.

156. Helmstaedter C, Pohl C, Elger CE (1995) Relations between verbal and nonverbal memory performance - Evidence of confounding effects particularly in patients with right temporal-lobe epilepsy. Cortex 31: 345-355.

157. Helmstaedter C, Sonntag-Dillender M, Hoppe C, Elger CE (2004) Depressed mood and memory impairment in temporal lobe epilepsy as a function of focus lateralization and localization. Epilepsy & Behavior 5: 696-701.

158. Hennenlotter A, Schroeder U, Erhard P, Haslinger B, Stahl R, et al. (2004) Neural correlates associated with impaired disgust processing in pre-symptomatic Huntington's disease. Brain 127: 1446-1453.

159. Henry M, Merten T, Wallasch TM (2008) Semantic dementia - A case report in the context of an independent medical examination. Fortschritte der Neurologie Psychiatrie 76: 453-464.

160. Hermann D, Sartorius A, Welzel H, Walter S, Skopp G, et al. (2007) Dorsolateral prefrontal cortex N-acetylaspartate/total creatine (NAA/tCr) loss in male recreational cannabis users. Biological Psychiatry 61: 1281-1289.

161. Hermelink K (2006) „Chemobrain“? Die kognitive Leistungsfähigkeit von Mammakarzinom-Patientinnen vor und während neoadjuvanter Chemotherapie ["Chemobrain"? Cognitive performance abilities of patients suffering from mammacarcinoma before and during neoadjuvant chemotherapy] [doctoral dissertation]. München: Ludwig-Maximilian-University München.

162. Herzog MH, Kopmann S, Brand A (2004) Intact figure-ground segmentation in schizophrenia. Psychiatry Research 129: 55-63.

163. Hildebrandt H, Brokate B, Eling P, Lanz M (2004) Response shifting and inhibition, but not working memory, are impaired after long-term heavy alcohol consumption. Neuropsychology 18: 203-211.

164. Hildebrandt H, Brokate B, Fink F, Muller SV, Eling P (2008) Impaired stimulus-outcome but preserved stimulus-response shifting in young substance-dependent individuals. Journal of Clinical and Experimental Neuropsychology 30: 946-955.

165. Hildebrandt H, Brokate B, Hoffmann E, Kroger B, Eling P (2006) Conditional responding is impaired in chronic alcoholics. Journal of Clinical and Experimental Neuropsychology 28: 631-645.

166. Hildebrandt H, Brokate B, Lanz M, Ternes T, Timm C (2003) Executive functions in patients with multiple sclerosis. Aktuelle Neurologie 30: 118-126.

167. Hildebrandt H, Clausing A, Janssen H, Mödden C (2007) Rehabilitation leichter bis mittelschwerer Gedächtnisdefizite—mehr Therapie hilft deutlich mehr, aber welche, wie und warum? [Rehabilitation of mild to moderately severe memory deficits—more therapy helps more, but which one, how, and why?]. Neurologie und Rehabilitation 13: 135-145.

168. Hodapp V, Sicker G, Wick AD, Winkelstrater R (1997) Anger and suicide risk. A study with older psychiatric patients. Nervenarzt 68: 55-61.

169. Hodl AK, Hodl E, Otti DV, Herranhof B, Ille R, et al. (2008) Ideomotor limb apraxia in Huntington's disease - A case-control study. Journal of Neurology 255: 331-339.

170. Hoenig K, Hochrein A, Muller DJ, Wagner M (2002) Different negative priming impairments in schizophrenia and subgroups of obsessive-compulsive disorder. Psychological Medicine 32: 459-468.

171. Hoenig K, Hochrein A, Quednow BB, Maier W, Wagner M (2005) Impaired prepulse inhibition of acoustic startle in obsessive-compulsive disorder. Biological Psychiatry 57: 1153-1158.

172. Hofer A, Baumgartner S, Bodner T, Edlinger M, Hummer M, et al. (2005) Patient outcomes in schizophrenia II: the impact of cognition. European Psychiatry 20: 395-402.

173. Hofer A, Niedermayer B, Kemmler G, Rettenbacher MA, Trebo E, et al. (2007) Cognitive impairment in schizophrenia: Clinical ratings are not a suitable alternative to neuropsychological testing. Schizophrenia Research 92: 126-131.

174. Hofer A, Rettenbacher MA, Edlinger M, Huber R, Bodner T, et al. (2007) Outcomes in schizophrenia outpatients treated with amisulpride or olanzapine. Pharmacopsychiatry 40: 1-8.

175. Hofleitner I (1983) Hirnleistungsdefizite und beeinflussende Störfaktoren bei Alkoholikern: Eine Untersuchung zur Erfassung der Verteilung von psychologischen, medizinischen und soziologischen Variablen in einer großen unselektierten Alkoholikergruppe, sowie zur Beschreibung des Einflusses dieser Variablen auf die Ergebnisse in speziellen Leistungstests [Deficits in brain performance and influencing factors in alcoholics: An investigation to assess the distribution of psychological, medical, and sociological variables in a big unselected group of alcoholics and a description of the influences of these variables on outcomes of special ability tasks] [doctoral thesis]. Vienna: University of Vienna.

176. Honegger J, Barocka A, Sadri B, Fahlbusch R (1998) Neuropsychological results of craniopharyngioma surgery in adults: A prospective study. Surgical Neurology 50: 19-28.

177. Hoppe C, Hoffmann J, Elger CE (2004) Immediate effects of intermittent high-amplitude vagus nerve stimulation ON phases on visuomotor reactions. Neurology Psychiatry and Brain Research 11: 179-184.

178. Hornstein C, Richter P, Mortimer A, Will A, Beuth A, et al. (1998) Dimensions of schizophrenia in the elderly: Correlations with cognitive and motor parameters. Nervenarzt 69: 243-248.

179. Hornung OP, Regen F, Warnstedt C, Anghelescu I, Danker-Hopfe H, et al. (2008) Declarative and procedural memory consolidation during sleep in patients with borderline personality disorder. Journal of Psychiatric Research 42: 653-658.

180. Hösch G (1995) Evaluation eines computergestützten Planspiels [Evaluation of a computer-based experimental-game] [doctoral dissertation]. Mainz: University of Mainz.

181. Huber A (1977) Ein Vergleich der Trennfähigkeit verschiedener Leistungs- und Selbstbeurteilungsverfahren bei endogen depressiven Patienten und Patienten mit Durchgangs-Syndrom [A comparison of the separability of different performance and self-assessment measures in endogenuos depressive patients with transition-syndrome] [doctoral dissertation]. Nürnberg: University of Erlangen-Nürnberg.

182. Hummel T, Barz S, Pauli E, Kobal G (1998) Chemosensory event-related potentials change with age. Electroencephalography and Clinical Neurophysiology 108: 208-217.

183. Hurlemann R, Jessen F, Wagner M, Frommann I, Ruhrmann S, et al. (2008) Interrelated neuropsychological and anatomical evidence of hippocampal pathology in the at-risk mental state. Psychological Medicine 38: 843-851.

184. Huwel J, Weisner B, Kemmer H, Heyder J (1998) Depression in the acute stage after the first ischemic stroke. Nervenarzt 69: 330-334.

185. Ihrig A, Dietz MC (2003) Use of psychological test battery in occupational prevention of neurotoxicity. Zeitschrift fur Arbeits- und Organisationspsychologie 47: 61-72.

186. Ihrig A, Dietz MC, Bader M, Triebig G (2005) Longitudinal study to explore chronic neuropsychologic effects on solvent exposed workers. Industrial Health 43: 588-596.

187. Ihrig A, Triebig G, Dietz MC (2001) Evaluation of a modified German version of the Q16 questionnaire for neurotoxic symptoms in workers exposed to solvents. Occupational and Environmental Medicine 58: 19-23.

188. Ille R, Lahousen T, Rous F, Hofmann R, Kapfhammer HP (2005) Personality profile and psychic deviations in offenders examined for psychiatric-forensic appraisal. Nervenarzt 76: 52-60.

189. Jager T, Kliegel M (2008) Time-based and event-based prospective memory across adulthood: underlying mechanisms and differential costs on the ongoing task. Journal of General Psychology 135: 4-22.

190. Jelinek L, Jacobsen D, Kellner M, Larbig F, Biesold KH, et al. (2006) Verbal and nonverbal memory functioning in posttraumatic stress disorder (PTSD). Journal of Clinical and Experimental Neuropsychology 28: 940-948.

191. Jelinek L, Moritz S, Randjbar S, Sommerfeldt D, Puschel K, et al. (2008) Does the evocation of traumatic memories confound subsequent working memory performance in posttraumatic stress disorder (PTSD)? Depression and Anxiety 25: 175-179.

192. Jockers-Scherubl MC, Wolf T, Radzei N, Schlattmann P, Rentzsch J, et al. (2007) Cannabis induces different cognitive changes in schizophrenic patients and in healthy controls. Progress in Neuro-Psychopharmacology & Biological Psychiatry 31: 1054-1063.

193. Jokeit H, Ebner A (2002) Effects of chronic epilepsy on intellectual functions. Progress in Brain Research 135: 455-463.

194. Jokeit H, Luerding R, Ebner A (2000) Cognitive impairment in temporal-lobe epilepsy. Lancet 355: 1018-1019.

195. Juckel G, Schlagenhauf F, Koslowski M, Wustenberg T, Villringer A, et al. (2006) Dysfunction of ventral striatal reward prediction in schizophrenia. Neuroimage 29: 409-416.

196. Junger A (1982) Zur Gültigkeit von selbstabnehmbaren psychometrischen und psychopathometrischen Verfahren in der zahnärztlichen Praxis: Vergleich von Trait- und State-Verfahren [Validity of self-assessable psychometric and psychopathometric measures in dental practice: A comparison of trait- and state-measures] [doctoral dissertation]. Nürnberg: University of Erlangen-Nürnberg.

197. Kaiser S, Roth A, Rentrop M, Friederich HC, Bender S, et al. (2008) Intra-individual reaction time variability in schizophrenia, depression and borderline personality disorder. Brain and Cognition 66: 73-82.

198. Kapoula O, Lehrl S, Fischer B, Burkard G, Schubak G (1990) Nimodipin bei Hirnleistungsstörungen im Alter: Eine placebokontrollierte Doppelblindstudie in ärztlichen Praxen [Nimodipin in brain performance disfunctions in old age: A placebo-controlled double-blind study in medical practices]. Geriatrie und Rehabilitation 3: 135-146.

199. Karch S, Graz C, Jager L, Karamatskos E, Stammel A, et al. (2007) Influence of anxiety on electrophysiological correlates of response inhibition capacities in alcoholism. Clinical EEG and Neuroscience 38: 89-95.

200. Karch S, Jager L, Karamatskos E, Graz C, Stammel A, et al. (2008) Influence of trait anxiety on inhibitory control in alcohol-dependent patients: Simultaneous acquisition of ERPs and BOLD responses. Journal of Psychiatric Research 42: 734-745.

201. Kaschel R (1994) Neuropsychologische Rehabilitation von Gedächtnisleistungen [Neuropsychological rehabilitation of brain performance]. Weinheim: Psychologie Verlags Union.

202. Kathmann N, Frodl-Bauch T, Hegerl U (1999) Stability of the mismatch negativity under different stimulus and attention conditions. Clinical Neurophysiology 110: 317-323.

203. Kathmann N, Hochrein A, Uwer R (1999) Effects of dual task demands on the accuracy of smooth pursuit eye movements. Psychophysiology 36: 158-163.

204. Kern I, Fischer U, Fischer B (1979) Correlation between the Mehrfachwahl-Wortschatz-Test (MWT-B) and the Progressive Matrices with pre-geriatric patients. Fortschritte der Medizin 97: 1821-1823.

205. Kessler J, Fast K, Mielke R (1995) The problems in diagnosing premorbid intelligence with the Multiple-Choice Word Test (MWT-B) in patients with Alzheimers-disease. Nervenarzt 66: 696-702.

206. Khatami R, Neumann M, Schulz H, Kolmel HW (1998) A family with autosomal dominant nocturnal frontal lobe epilepsy and mental retardation. Journal of Neurology 245: 809-810.

207. Kick HA (1991) Psychopathologie und Verlauf der postakuten Schizophrenie [Psychopathology and devolution of postacute schizophrenia]. Berlin: Springer.

208. Kiesswetter E, Sietmann B, Zupanic M, Seeber A (2000) Neurobehavioral study on the interactive effects of age and solvent exposure. Neurotoxicology 21: 685-695.

209. King JA, Colla M, Brass M, Heuser I, von Cramon DY (2007) Inefficient cognitive control in adult ADHD: evidence from trial-by-trial Stroop test and cued task switching performance. Behavioral and Brain Functions 3: 42.

210. Kinzel W, Galster JV, Erzigkeit H, Lamprecht W (1979) Is there a significant correlation between the degree of personality changes following severe head-injuries and intelligence. Fortschritte der Neurologie Psychiatrie 47: 67-83.

211. Kircher T, Whitney C, Krings T, Huber W, Weis S (2008) Hippocampal dysfunction during free word association in male patients with schizophrenia. Schizophrenia Research 101: 242-255.

212. Kircher TT, Seiferth NY, Plewnia C, Baar S, Schwabe R (2007) Self-face recognition in schizophrenia. Schizophrenia Research 94: 264-272.

213. Kircher TTJ, Koch K, Stottmeister F, Durst V (2007) Metacognition and reflexivity in patients with schizophrenia. Psychopathology 40: 254-260.

214. Kircher TTJ, Leube DT, Erb M, Grodd W, Rap AM (2007) Neural correlates of metaphor processing in schizophrenia. Neuroimage 34: 281-289.

215. Kliegel M, Altgassen M (2006) Interindividual differences in learning performance: The effects of age, intelligence, and strategic task approach. Educational Gerontology 32: 111-124.

216. Kliegel M, Altgassen M, Martin M, Kruse A (2003) Learning in old age: The importance of self-initiated structuring. Zeitschrift fur Gerontologie und Geriatrie 36: 421-428.

217. Kliegel M, Eschen A, Thone-Otto AIT (2004) Planning and realization of complex intentions in traumatic brain injury and normal aging. Brain and Cognition 56: 43-54.

218. Kliegel M, Jaeger T (2006) Delayed-execute prospective memory performance: The effects of age and working memory. Developmental Neuropsychology 30: 819-843.

219. Kliegel M, Jager T (2006) Development of prospective memory across the lifespan. Zeitschrift fur Entwicklungspsychologie und Padagogische Psychologie 38: 162-174.

220. Kliegel M, Martin M (2003) Prospective memory research: Why is it relevant? International Journal of Psychology 38: 193-194.

221. Kliegel M, Phillips LH, Lemke U, Kopp UA (2005) Planning and realisation of complex intentions in patients with Parkinson's disease. Journal of Neurology Neurosurgery and Psychiatry 76: 1501-1505.

222. Kliegl R, Grabner E, Rolfs M, Engbert R (2004) Length, frequency, and predictability effects of words on eye movements in reading. European Journal of Cognitive Psychology 16: 262-284.

223. Klieser E, Lehmann E (1993) Wovon hängt der Mißerfolg der Psychopharmakotherapie ab? [On what depends failure of psychopharmacotherapy]. In: Hinterhuber H, Kulhanek F, Fleischhacker WW, Neumann R, editors. Prädiktoren und Therapieresistenz in der Psychiatrie [Predictors and resistance to therapy in psychiatry]. Braunschweig: Vieweg. pp. 146-151.

224. Klosak S, Bühler KE (1999) Psychometrische Differenzierung von Schizophrenen, schizoaffektiven, depressiven und alkoholabhängigen Patienten [Psychometric differentiation of schizophrenic, schizoaffective, depressive, and alcohol-dependent patients]. Schweizer Archiv für Neurologie und Psychiatrie 150: 238-247.

225. Knauper B, Wittchen HU (1994) Diagnosing major depression in the elderly - Evidence for response bias in standardized diagnostic interviews. Journal of Psychiatric Research 28: 147-164.

226. Knoblich G, Stottmeister F, Kircher T (2004) Self-monitoring in patients with schizophrenia. Psychological Medicine 34: 1561-1569.

227. Knopf M (1995) Beyond verbal memory: Enhancing memory by acting. In: Vellas BJ, Albarede JL, Garry PJ, editors. Mood and cognitive disorders: Facts and research in gerontology. New York: Springer. pp. 43-53.

228. Koch K, Pauly K, Kellermann T, Seiferth NY, Reske M, et al. (2007) Gender differences in the cognitive control of emotion: An fMRI study. Neuropsychologia 45: 2744-2754.

229. Kockelmann E, Elger CE, Hehnstaedter C (2003) Significant improvement in frontal lobe associated neuropsychological functions after withdrawal of Topiramate in epilepsy patients. Epilepsy Research 54: 171-178.

230. Kockelmann E, Elger CE, Helmstaedter C (2004) Cognitive profile of topiramate as compared with lamotrigine in epilepsy patients on antiepileptic drug polytherapy: relationships to blood serum levels and comedication. Epilepsy & Behavior 5: 716-721.

231. Konczalski H, Kujumdshiev G, Meyer-Probst B, Schwanewede HV (1987) The role of psychogenic factors in intolerance to prosthetics. Zahn- Mund- und Kieferheilkunde 75: 31-36.

232. Konrad C, Engelien A, Schoning S, Zwitserlood P, Jansen A, et al. (2008) The functional anatomy of semantic retrieval is influenced by gender, menstrual cycle, and sex hormones. Journal of Neural Transmission 115: 1327-1337.

233. Kopp B (2007) Mnemonic intrusions into working memory in psychometrically identified schizotypal individuals. Journal of Behavior Therapy and Experimental Psychiatry 38: 56-74.

234. Kopp B, Wolff M, Hruska C, Reischies FM (2002) Brain mechanisms of visual encoding and working memory in psychometrically identified schizotypal individuals and after acute administration of haloperidol. Psychophysiology 39: 459-472.

235. Kopp UA, Thone-Otto AIT (2003) Disentangling executive functions and memory processes in event-based prospective remembering after brain damage: A neuropsychological study. International Journal of Psychology 38: 229-235.

236. Kraemer S, Schickor I (1991) Stressbewältigungsstrategien schizophrener Patienten: Eine Pilotstudie [Stress coping strategies of schizophrenic patients: A pilot study]. Verhaltenstherapie 1: 212-218.

237. Kray J, Eppinger B (2006) Effects of associative learning on age differences in task-set switching. Acta Psychologica 123: 187-203.

238. Kray J, Eppinger B, Mecklinger A (2005) Age differences in attentional control: An event-related potential approach. Psychophysiology 42: 407-416.

239. Krug A, Markov V, Eggermann T, Krach S, Zerres K, et al. (2008) Genetic variation in the schizophrenia-risk gene neuregulin1 correlates with differences in frontal brain activation in a working memory task in healthy individuals. Neuroimage 42: 1569-1576.

240. Kuelz AK, Riemann D, Halsband U, Vielhaber K, Unterrainer J, et al. (2006) Neuropsychological impairment in obsessive-compulsive disorder - Improvement over the course of cognitive behavioral treatment. Journal of Clinical and Experimental Neuropsychology 28: 1273-1287.

241. Kuelz AK, Riemann D, Zahn R, Voderholzer U (2004) Object alternation test - Is it sensitive enough to detect cognitive dysfunction in obsessive-compulsive disorder? European Psychiatry 19: 441-443.

242. Kugler CFA (1999) Interrelations of age, sensory functions, and human brain signal processing. Journals of Gerontology Series A-Biological Sciences and Medical Sciences 54: 231-238.

243. Kukolja J, Schlapfer TE, Keysers C, Klingmuller D, Maier W, et al. (2008) Modeling a negative response bias in the human amygdala by noradrenergic-glucocorticoid interactions. Journal of Neuroscience 28: 12868-12876.

244. Kurscheidt JC, Peiler P, Behnken A, Abel S, Pedersen A, et al. (2008) Acute effects of methylphenidate on neuropsychological parameters in adults with ADHD: Possible relevance for therapy. Journal of Neural Transmission 115: 357-362.

245. Labudda K, Todorovski S, Markowitsch HJ, Brand M (2008) Judgment and memory performance for emotional stimuli in patients with alcoholic Korsakoff syndrome. Journal of Clinical and Experimental Neuropsychology 30: 224-235.

246. Ladurner G, Holzer H, Wawschinek O, Pogglitsch H, Petek W (1981) The Importance of Aluminum in Dialyseencephalopathie. Fortschritte der Neurologie Psychiatrie 49: 211-213.

247. Lagois M (1976) Versuch der Differenzierung von funktionspsychotischen, schizophrenen, zyklothym depressiven Patienten und psychiatrischen Kontrollpersonen durch Tests für flüssige und kristallisierte Intelligenz, sowie Kreativität [Attempt of a differentiation of functional psychotic, cyclothymic depressive patients and psychiatric control subjects by tests for fluid and crystallized intelligence as well as creativity] [doctoral thesis]. Nürnberg: University of Erlangen-Nürnberg.

248. Lamm C, Bauer H, Vitouch O, Gstattner R (1999) Differences in the ability to process a visuo-spatial task are reflected in event-related slow cortical potentials of human subjects. Neuroscience Letters 269: 137-140.

249. Lamm C, Fischmeister FPS, Bauer H (2005) Individual differences in brain activity during visuo-spatial processing assessed by slow cortical potentials and LORETA. Cognitive Brain Research 25: 900-912.

250. Lamm C, Windischberger C, Leodolter U, Moser E, Bauer H (2001) Evidence for premotor cortex activity during dynamic visuospatial imagery from single-trial functional magnetic resonance imaging and event-related slow cortical potentials. Neuroimage 14: 268-283.

251. Lang C, Lehrl S, Huk W (1981) A Case of bilateral temporal-lobe agenesis. Journal of Neurology Neurosurgery and Psychiatry 44: 626-630.

252. Lang CJG, Majer M, Balan P, Reischies FM (2000) Recall and recognition in Huntington's disease. Archives of Clinical Neuropsychology 15: 361-371.

253. Lang CJG, Reischies FM, Majer M, Daum RF (1999) Visually guided exploration in Huntington disease. Cortex 35: 583-590.

254. Lange KW, Tucha O, Alders GL, Preier M, Csoti I, et al. (2003) Differentiation of parkinsonian syndromes according to differences in executive functions. Journal of Neural Transmission 110: 983-995.

255. Lehrl S (1980) Einfluß vergangener und akuter Krankenhausaufenthalte auf fluide und kristallisierte Intelligenzleistungen [Influences of past and acute hospitalization on fluid and cristallized intelligence test performance]. Vaterstetten-München: Vless.

256. Lehrl S (1981) Hatte Francis Galton doch recht? Informationspsychologischer Beitrag zur Verteilung intellektueller Begabungen [Was Galton right? Informationpsychological contribution to the distribution of intellectual talents]. Grundlagenstudien aus Kybernetik und Geisteswissenschaft 22: 17-28.

257. Lehrl S, Cziske R, Fischer B (1981) „Dosis“ bedeutet: Verpackung in der Dose [„Dosage“ means: Wrapping in cans]. Moderne Medizin 9: 1228-1239.

258. Lehrl S, Fischer B, Dickreiter B (1989) Pentoxifyllin und Co-Dergocrinmesilat bei ätiopathogenetisch unausgelesenen Patienten mit zerebraler Insuffizienz: Ein psychopathometrischer Doppelblind-Vergleich [Pentoxifyllin and Co-Dergocrinmesilat in etiopathogenial unselected patients with cerebral insufficiency: A double-blind comparison]. Geriatrie und Rehabilitation 2: 59-70.

259. Lehrl S, Fischer B, Weidenhammer W, Schmidt A, Upmeyer H-J (1988) Laienwissen und AIDS-Aufklärungskampagnen [Laymens knowledge and AIDS-information campaigns]. In: Lehrl S, Kinzel W, Fischer B, editors. Psychopathometrie in der Medizin: Beispiele und Ergebnisse von Anwendungen in Forschung und Praxis [Psychopathometrics in medicine: Examples and results of applications in research and practice]. Ebersberg: Vless. pp. 29-43.

260. Lehrl S, Roth FG (1973) Relations between subjected classification of pain and intelligence, age and mood. Arzneimittel-Forschung/Drug Research 23: 999-1002.

261. Lehrner J, Eckersberger C, Walla P, Potsch G, Deecke L (2000) Ambient odor of orange in a dental office reduces anxiety and improves mood in female patients. Physiology & Behavior 71: 83-86.

262. Lehrner J, Gleiss A, Maly J, Auff E, Dal-Bianco P (2006) The Verbal Selective Reminding Test (VSRT). Neuropsychiatrie 20: 204-214.

263. Lehrner J, Gufler R, Guttmann G, Maly J, Gleiss A, et al. (2005) Annual conversion to Alzheimer disease among patients with memory complaints attending an outpatient memory clinic: The influence of amnestic mild cognitive impairment and the predictive value of neuropsychological testing. Wiener Klinische Wochenschrift 117: 629-635.

264. Lehrner J, Kalchmayr R, Serles W, Olbrich A, Pataraia E, et al. (1999) Health-related quality of life (HRQOL), activity of daily living (ADL) and depressive mood disorder in temporal lobe epilepsy patients. Seizure-European Journal of Epilepsy 8: 88-92.

265. Lehrner J, Willfort A, Mlekusch I, Guttmann G, Minar E, et al. (2005) Neuropsychological outcome 6 months after unilateral carotid stenting. Journal of Clinical and Experimental Neuropsychology 27: 859-866.

266. Lendt M, Helmstaedter C, Elger CE (1997) Pre- and postoperative socioeconomic development of 151 patients with focal epilepsies. Epilepsia 38: 1330-1337.

267. Leplow B, Dierks C (1997) Gilt das Gesetz von Ribot auch für das Altgedächtnis? [Does the law of Ribot apply to the remote memory?]. Zeitschrift für Differentielle und Diagnostische Psychologie 18: 215-226.

268. Leplow B, Dierks C (1997) Assessment of remote memory by means of a German ''famous events test''. Diagnostica 43: 193-209.

269. Leplow B, Dierks C, Herrmann P, Pieper N, Annecke R, et al. (1997) Remote memory in Parkinson's disease and senile dementia. Neuropsychologia 35: 547-557.

270. Leplow B, Dierks C, Merten T, Hänsgen K (1997) Probleme des Geltungsbereichs deutschsprachiger Altgedächtnistest [Problems of the scope of the German remote memory test]. Zeitschrift für Neuropsychologie 8: 137-144.

271. Leplow B, Dierks CH, Lehnung M, Kenkel S, Behrens C, et al. (1997) Remote memory in patients with acute brain injuries. Neuropsychologia 35: 881-892.

272. Leplow B, Friege L (1998) A demographically based index for the estimation of premorbid intelligence. Zeitschrift fur Klinische Psychologie-Forschung und Praxis 27: 1-8.

273. Leplow B, Holl D, Zeng LJ, Herzog A, Behrens K, et al. (2002) Spatial behaviour is driven by proximal cues even in mildly impaired Parkinson's disease. Neuropsychologia 40: 1443-1455.

274. Leplow B, Murphy R, Nutzinger DO (2002) Specificity of conditional associative-learning deficits in obsessive-compulsive disorder (OCD) and non-OCD anxiety disorders. Journal of Clinical and Experimental Neuropsychology 24: 792-805.

275. Leplow B, Tetzlaff K, Holl D, Zeng L, Reuter M (2001) Spatial orientation in construction divers - are there associations with diving experience? International Archives of Occupational and Environmental Health 74: 189-198.

276. Leppert K (1998) Kognitive Leistungsfähigkeit und kognitiver Stil bei Patienten mit Typ-II-Diabetes zum Schulungszeitpunkt: Implikationen für das strukturierte Behandlungs- und Schulungsprogramm für konventionelle Insulintherapie bei älteren Typ-II-Diabetikern [Cognitive capabilities and cognitive style of patients suffering of type-II-diabetes at training: Implications of structured treatment and training programs for conventional insulin therapy in elderly subjects suffering from type-II-diabetes] [doctoral dissertation]. Jena: University of Jena.

277. Leyhe T, Mussig K, Weinert C, Laske C, Haring HU, et al. (2008) Increased occurrence of weaknesses in attention testing in patients with Hashimoto's thyroiditis compared to patients with other thyroid illnesses. Psychoneuroendocrinology 33: 1432-1436.

278. Linzmayer L, Semlitsch HV, Saletu B, Bock G, Saletu-Zyhlarz G, et al. (2001) Double-blind, placebo-controlled psychometric studies on the effects of a combined estrogen-progestin regimen versus estrogen alone on performance, mood and personality of menopausal syndrome patients. Arzneimittel-Forschung-Drug Research 51: 238-245.

279. Littmann E (1981) Results of psychodiagnostic examinations in forensic-psychologically and psychiatrically evaluated criminals. Psychiatrie, Neurologie und Medizinische Psychologie 33: 734-743.

280. Littmann E (1985) Zur Persönlichkeitsstruktur forensisch begutachteter Sexualstraftäter [Personality structure of forensically examined sexuall offenders]. In: Jähnig H-U, Littmann E, editors. Kriminalpsychologie und Kriminalpsychopathologie [Criminal psychology and criminal psychopathology]. Jena: Gustav Fischer. pp. 147-165.

281. Littmann E, Friemert K, Szewczyk H (1989) Abnormal psychosocial development and legal responsibility--results of psychopathometric studies. Psychiatrie, Neurologie und Medizinische Psychologie 41: 269-279.

282. Loeber S, Kniest A, Diehl A, Mann K, Croissant B (2008) Neuropsychological functioning of opiate-dependent patients: A nonrandomized comparison of patients preferring either buprenorphine or methadone maintenance treatment. American Journal of Drug and Alcohol Abuse 34: 584-593.

283. Lux S, Helmstaedter C, Elger CE (1999) Normative study on the "Verbaler Lern- und Merkfahigkeitstest" (VLMT). Diagnostica 45: 205-211.

284. Mann K, Ackermann K, Croissant B, Mundle G, Nakovics H, et al. (2005) Neuroimaging of gender differences in alcohol dependence: Are women more vulnerable? Alcoholism-Clinical and Experimental Research 29: 896-901.

285. Mann K, Gunther A, Stetter F, Ackermann K (1999) Rapid recovery from cognitive deficits in abstinent alcoholics: A controlled test-retest study. Alcohol and Alcoholism 34: 567-574.

286. Markowitsch HJ, Fink GR, Thöne A, Kessler J, Heiss W-D (1997) A PET study of persistent psychogenic amnesia covering the whole life span. Cognitive Neuropsychiatry 2: 135-158.

287. Markowitsch HJ, Kessler J, Russ MO, Frolich L, Schneider B, et al. (1999) Mnestic block syndrome. Cortex 35: 219-230.

288. Markowitsch HJ, Von Cramon DY, Schuri U (1993) Mnestic performance profile of a bilateral diencephalic infarct patient with preserved intelligence and severe amnesic disturbances. Journal of Clinical and Experimental Neuropsychology 15: 627-652.

289. Markowitsch HJ, WeberLuxemburger G, Ewald K, Kessler J, Heiss WD (1997) Patients with heart attacks are not valid models for medial temporal lobe amnesia. A neuropsychological and FDG-PET study with consequences for memory research. European Journal of Neurology 4: 178-184.

290. Mark-Stemberger B (1988) Suizidales Verhalten: Lerntheoretische Aspekte von Wiederholungstendenzen [Suicidal behavior: Learn theoretical aspects of recurrence tendencies] [doctoral thesis]. Innsbruck: University of Innsbruck.

291. Martin A, Buech A, Schwenk C, Rief W (2007) Memory bias for health-related information in somatoform disorders. Journal of Psychosomatic Research 63: 663-671.

292. Martin M, Ewert O (1996) Problem solving in older adults: A German adaptation of the Everyday Problem Solving Inventory. Zeitschrift fur Entwicklungspsychologie und Padagogische Psychologie 28: 380-399.

293. Martin M, Ewert O (1997) Attention and planning in older adults. International Journal of Behavioral Development 20: 577-594.

294. Martin M, Schumann-Hengsteler R (1996) Aging and performance in different prospective memory measures. Zeitschrift fur Gerontologie und Geriatrie 29: 119-126.

295. Martin M, Schumann-Hengsteler R (2001) How task demands influence time-based prospective memory performance in young and older adults. International Journal of Behavioral Development 25: 386-391.

296. Mavrogiorgou P, Mergl R, Tigges P, El Husseini J, Schroter A, et al. (2001) Kinematic analysis of handwriting movements in patients with obsessive-compulsive disorder. Journal of Neurology Neurosurgery and Psychiatry 70: 605-612.

297. Mayr U (2001) Age differences in the selection of mental sets: The role of inhibition, stimulus ambiguity, and response-set overlap. Psychology and Aging 16: 96-109.

298. Meisenzahl EM, Koutsouleris N, Gaser C, Bottlender R, Schmitt GJE, et al. (2008) Structural brain alterations in subjects at high-risk of psychosis: A voxel-based morphometric study. Schizophrenia Research 102: 150-162.

299. Mell T, Heekeren HR, Marschner A, Wartenburger I, Villringer A, et al. (2005) Effect of aging on stimulus-reward association learning. Neuropsychologia 43: 554-563.

300. Mergl R, Tigges P, Schroter A, Moller HJ, Hegerl U (1999) Digitized analysis of handwriting and drawing movements in healthy subjects: methods, results and perspectives. Journal of Neuroscience Methods 90: 157-169.

301. Merkelbach S, Sittinger H, Schweizer I, Muller M (2000) Cognitive outcome after bacterial meningitis. Acta Neurologica Scandinavica 102: 118-123.

302. Merten T (1992) Word association and schizophrenia - An empirical study. Nervenarzt 63: 401-408.

303. Merten T (1995) Factors influencing word association responses - A reanalysis. Creativity Research Journal 8: 249-263.

304. Merten T, Fischer I (1999) Creativity, personality and word association responses: associative behaviour in forty supposedly creative persons. Personality and Individual Differences 27: 933-942.

305. Messerklinger H, Marx R (1989) Die Restitution feinmotorischer Leistungsparameter bei chronischen Alkoholikern während einer 6-wöchigen Entzugsbehandlung unter besonderer Berücksichtigung des Residualsyndroms [Restitution of fine motor skill parameters in chronic alcoholics during a 6 week period of deprivation treatment under consideration of the residual syndrome]. Wiener Zeitschrift für Suchtforschung 12: 21-38.

306. Metzler P, Haas W, Potel C (2002) Memory disturbances following unilateral cerebral lesions. Nervenarzt 73: 355-363.

307. Michelfelder H (1998) Multimodale Intervention auf der Basis eines Gedächtnistrainings mit älteren Menschen [Multimodal intervention on basis of memory training in elderly persons]. Frankfurt/Main: Lang.

308. Mokros A, Menner B, Eisenbarth H, Alpers GW, Lange KW, et al. (2008) Diminished cooperativeness of psychopaths in a prisoner's dilemma game yields higher rewards. Journal of Abnormal Psychology 117: 406-413.

309. Montag C, Heinz A, Kunz D, Gallinat J (2007) Self-reported empathic abilities in schizophrenia. Schizophrenia Research 92: 85-89.

310. Moritz S, Andresen B, Jacobsen D, Mersmann K, Wilke U, et al. (2001) Neurokognitive Korrelate des Drei-Faktoren-Modells der Schizophrenie und Schizotypie: Evaluation eines Instrumentes zur Erfassung schizophrener Symptomatik (PANADSS) [Neurocognitive correlates of the three-factor-model of schizophrenia and schizotypy: Evaluation of a measure to assess schizophrenic symptomatic]. In: Andresen B, Mass R, editors. Schizotypie: Psychometrische Entwicklungen und biopsychologische Forschungsansätze [Schizotypy: Psychometric developments and biopsychological research approaches]. Göttingen: Hogrefe. pp. 569-582.

311. Moritz S, Andresen B, Jacobsen D, Mersmann K, Wilke U, et al. (2001) Neuropsychological correlates of schizophrenic syndromes in patients treated with atypical neuroleptics. European Psychiatry 16: 354-361.

312. Moritz S, Andresen B, Perro C, Schickel M, Krausz M, et al. (2002) Neurocognitive performance in first-episode and chronic schizophrenic patients. European Archives of Psychiatry and Clinical Neuroscience 252: 33-37.

313. Moritz S, Birkner C, Kloss M, Jahn H, Hand I, et al. (2002) Executive functioning in obsessive-compulsive disorder, unipolar depression, and schizophrenia. Archives of Clinical Neuropsychology 17: 477-483.

314. Moritz S, Hubner M, Kluwe R (2004) Task switching and backward inhibition in obsessive-compulsive disorder. Journal of Clinical and Experimental Neuropsychology 26: 677-683.

315. Moritz S, Iverson GL, Woodward TS (2003) Reliable change indexes for memory performance in schizophrenia as a means to determine drug-induced cognitive decline. Applied Neuropsychology 10: 115-120.

316. Moritz S, Meier B, Hand I, Schick M, Jahn H (2004) Dimensional structure of the Hamilton Depression Rating Scale in patients with obsessive-compulsive disorder. Psychiatry Research 125: 171-180.

317. Moritz S, von Muhlenen A (2005) Inhibition of return in patients with obsessive-compulsive disorder. Journal of Anxiety Disorders 19: 117-126.

318. Moritz S, Woodward TS (2006) A generalized bias against disconfirmatory evidence in schizophrenia. Psychiatry Research 142: 157-165.

319. Moritz S, Woodward TS, Jelinek L, Klinge R (2008) Memory and metamemory in schizophrenia: a liberal acceptance account of psychosis. Psychological Medicine 38: 825-832.

320. Moritz S, Woodward TS, Lambert M (2007) Under what circumstances do patients with schizophrenia jump to conclusions? A liberal acceptance account. British Journal of Clinical Psychology 46: 127-137.

321. Moritz S, Woodward TS, Ruff CC (2003) Source monitoring and memory confidence in schizophrenia. Psychological Medicine 33: 131-139.

322. Mueller CA, Grassinger E, Naka A, Temmel AFP, Hummel T, et al. (2006) A self-administered odor identification test procedure using the "Sniffin' Sticks". Chemical Senses 31: 595-598.

323. Muller BW, Gimbel K, Keller-Pliessnig A, Sartory G, Gastpar M, et al. (2007) Neuropsychological assessment of adult patients with attention-deficit/hyperactivity disorder. European Archives of Psychiatry and Clinical Neuroscience 257: 112-119.

324. Muller U, Wachter T, Barthel H, Reuter M, von Cramon DY (2000) Striatal [I-123]beta-CIT SPECT and prefrontal cognitive functions in Parkinson's disease. Journal of Neural Transmission 107: 303-319.

325. Muller-Vahl KR, Koblenz A, Jobges M, Kolbe H, Emrich HM, et al. (2001) Influence of treatment of Tourette syndrome with delta(9)-tetrahydrocannabinol (delta(9)-THC) on neuropsychological performance. Pharmacopsychiatry 34: 19-24.

326. Mundt C, Barnett W, Witt G (1995) The core of negative symptoms in schizophrenia - Affect or cognitive deficiency. Psychopathology 28: 46-54.

327. Murphy R, Nutzinger DO, Paul T, Leplow B (2002) Dissociated conditional-associative learning in anorexia nervosa. Journal of Clinical and Experimental Neuropsychology 24: 176-186.

328. Murphy R, Nutzinger DO, Paul T, Leplow B (2004) Conditional-associative learning in eating disorders: A comparison with OCD. Journal of Clinical and Experimental Neuropsychology 26: 190-199.

329. Mussell M, Hewer W, Kulzer B, Bergis K, Rist F (2004) Effects of improved glycaemic control maintained for 3 months on cognitive function in patients with Type 2 diabetes. Diabetic Medicine 21: 1253-1256.

330. Naber D, Perro C, Schick U, Sadri I, Schmauss M, et al. (1989) Psychiatric-Symptoms and Neuropsychological Abnormalities in Hiv Infection. Nervenarzt 60: 80-85.

331. Nasterlack M, Dietz MC, Frank KH, Hacke W, Scherg H, et al. (1999) A multidisciplinary cross-sectional study on solvent-related health effects in painters compared with construction workers. International Archives of Occupational and Environmental Health 72: 205-214.

332. Netzer C, Helmstaedter C, Ehrbrecht A, Engels H, Schwanitz G, et al. (2006) Global brain dysmyelination with above-average verbal skills in 18q-syndrome with a 17 Mb terminal deletion. Acta Neurologica Scandinavica 114: 133-138.

333. Neudert C, Wasner M, Borasio GD (2001) Patients' assessment of quality of life instruments: a randomised study of SIP, SF-36 and SEIQoL-DW in patients with amyotrophic lateral sclerosis. Journal of the Neurological Sciences 191: 103-109.

334. Neuhaus AH, Koehler S, Opgen-Rhein C, Urbanek C, Hahn E, et al. (2007) Selective anterior cingulate cortex deficit during conflict solution in schizophrenia: An event-related potential study. Journal of Psychiatric Research 41: 635-644.

335. Neumann J, Wolfram H (1978) Application of the multiple vocabulary test to determining premorbid intelligence levels. Psychiatrie, Neurologie und Medizinische Psychologie 30: 721-730.

336. Neumann J, Wolfram H (1978) Application of the multiple vocabulary test to assessing mental disorders. Psychiatrie, Neurologie und Medizinische Psychologie 30: 731-738.

337. Neuser J (1983) Der Syndrom-Kurztest (SKT) als reliable Methode zur Erfassung des schweren Durchgangs-Syndroms [The syndrome-short-test (SKT) as a reliable measure to assess the transition sydrome] [doctoral dissertation]. Nürnberg: University of Erlangen-Nürnberg.

338. Nyffeler T, Gutbrod K, Pflugshaupt T, von Wartburg R, Hess CW, et al. (2005) Allocentric and egocentric spatial impairments in a case of topographical disorientation. Cortex 41: 133-143.

339. Oberauer K (2005) Control of the contents of working memory - A comparison of two paradigms and two age groups. Journal of Experimental Psychology-Learning Memory and Cognition 31: 714-728.

340. Oblak G, Egger JW (1996) Veränderungen in Befindlichkeits- und neuropsychologischen Leistungsvariablen durch Carotisendarterectomie bei symptomatischen und asymptomatischen Patienten [Changes in affectivity and neuropsychological performance variables due to carotisendarterectomy in symptomatic and nonsymptomatic patients]. Psychologie in der Medizin 7: 14-17.

341. Ohlmeier MD, Jahn K, Wilhelm-Gossling C, Godecke-Koch T, Hoffmann J, et al. (2007) Perazine and carbamazepine in comparison to olanzapine in schizophrenia. Neuropsychobiology 55: 81-88.

342. Opgenoorth E, Baldaszti E, Voracek M (1996) Viennese versions for the clinical application of the Mehrfachwahl-Wortschatz-Intelligenztest (MWT-A, MWT-B). Wiener Klinische Wochenschrift 108: 59-68.

343. Opgen-Rhein C, Neuhaus AH, Urbanek C, Hahn E, Sander T, et al. (2008) Executive attention in schizophrenic males and the impact of COMT Val(108/158)Met genotype on performance on the Attention Network Test. Schizophrenia Bulletin 34: 1231-1239.

344. Ott C (1996) Alkoholismus: Morphologische und psychopathologische Aspekte eines mehrdimensionalen Problems [Alcoholism: Morpholigical and psychopathological aspects of a multidimensional problem]. Regensburg: Roderer.

345. Ott G, Gottwald W, Ott C (1988) Psychopathometrische und psychopathologische Untersuchungen an Patienten mit Glossodynie [Psychopathometrical and psychopathological examinations of patients suffering from glossodyny]. In: Lehrl S, Kinzel W, Fischer B, editors. Psychopathometrie in der Medizin: Beispiele und Ergebnisse von Anwendungen in Forschung und Praxis [Psychopathometry in medicin: Examples and results of applications in research and practice]. Ebersberg: Vless. pp. 50-57.

346. Paelecke-Habermann Y, Pohl J, Leplow B (2005) Attention and executive functions in remitted major depression patients. Journal of Affective Disorders 89: 125-135.

347. Pakesch G, Pfersmann D, Loimer N, Grunberger J, Linzmayer L, et al. (1992) Neuropsychological assessment and psychopathological status in HIV-1 patients of different risk groups. Fortschritte der Neurologie Psychiatrie 60: 17-27.

348. Pater W (1979) Die Erlanger-Bilder-Skala (EBS): Ein psychopathometrischer Kurztest zur Quantifizierung der Intelligenzschwäche Erwachsener [The Erlanger-picture-scale (EBS): A psychopathometric shorttest for the quantification of adult intellectual weaknesses] [doctoral disseration]. Nürnberg: University of Erlangen-Nürnberg.

349. Pauly K, Seiferth NY, Kellermann T, Backes V, Vloet TD, et al. (2008) Cerebral dysfunctions of emotion-cognition interactions in adolescent-onset schizophrenia. Journal of the American Academy of Child and Adolescent Psychiatry 47: 1299-1310.

350. Pedersen A, Siegmund A, Ohrmann P, Rist F, Rothermundt M, et al. (2008) Reduced implicit and explicit sequence learning in first-episode schizophrenia. Neuropsychologia 46: 186-195.

351. Petrovsky N, Weiss-Motz F, Schulze-Rauschenbach S, Lemke M, Hornung P, et al. (2008) Antisaccade performance is related to genetic loading for schizophrenia. Journal of Psychiatric Research 43: 291-297.

352. Pfister E, Bockelmann I, Darius S, Wurthmann C (1999) Diagnosis of early toxic effects of lead or mixed organic solvents - Inclusion of psychopathological methods. Fortschritte der Neurologie Psychiatrie 67: 435-440.

353. Pfleger U (1996) Computerunterstütztes kognitives Trainingsprogramm mit schizophrenen Patienten [Computer-assisted cognitive training program for schizophrenic patients]. Münster: Waxmann.

354. Pietschmann M, Simon K, Endrass T, Kathmann N (2008) Changes of performance monitoring with learning in older and younger adults. Psychophysiology 45: 559-568.

355. Piringer G (1986) Werthaltungen und Persönlichkeitsmerkmale bei Myocardinfarkt- und Asthma-Bronchiale-Patienten [Principles and personality characteristics of myocardial infarction and asthma patients] [doctoral dissertation]. Salzburg: University of Salzburg.

356. Plichta MM, Vasic N, Wolf RC, Lesch KP, Brummer D, et al. (2009) Neural hyporesponsiveness and hyperresponsiveness during immediate and delayed reward processing in adult attention-deficit/hyperactivity disorder. Biological Psychiatry 65: 7-14.

357. Poppelreuter M, Weis J, Kulz AK, Tucha O, Lange KW, et al. (2004) Cognitive dysfunction and subjective complaints of cancer patients: a cross-sectional study in a cancer rehabilitation centre. European Journal of Cancer 40: 43-49.

358. Ptucha J, Hieber K (2000) „Typisch Knacki?“ – Typen Delinquenter in Thüringen: Resultate einer Jahreserhebung zu Persönlichkeit und Delinquenz im Thüringer Jugendstrafvollzug ["Typically jailbird?" - Types of delinquents in Thuringia: Results of an assessment over one year of personality and delinquency in the Thuringian correctional facilities for youths]. Zeitschrift für Strafvollzug und Straffälligenhilfe 49: 27-30.

359. Pukrop R, Matuschek E, Ruhrmann S, Brockhaus-Dumke A, Tendolkar I, et al. (2003) Dimensions of working memory dysfunction in schizophrenia. Schizophrenia Research 62: 259-268.

360. Pukrop R, Ruhrmann S, Schultze-Lutter F, Bechdolf A, Brockhaus-Dumke A, et al. (2007) Neurocognitive indicators for a conversion to psychosis: Comparison of patients in a potentially initial prodromal state who did or did not convert to a psychosis. Schizophrenia Research 92: 116-125.

361. Pukrop R, Schultze-Lutter F, Ruhrmann S, Brockhaus-Dumke A, Tendolkar I, et al. (2006) Neurocognitive functioning in subjects at risk for a first episode of psychosis compared with first- and multiple-episode schizophrenia. Journal of Clinical and Experimental Neuropsychology 28: 1388-1407.

362. Quednow BB, Jessen F, Kuhn KU, Maier W, Daum I, et al. (2006) Memory deficits in abstinent MDMA (ecstasy) users: Neuropsychological evidence of frontal dysfunction. Journal of Psychopharmacology 20: 373-384.

363. Quednow BB, Kuehn KU, Hoppe C, Westheide J, Maier W, et al. (2007) Elevated impulsivity and impaired decision-making cognition in heavy users of MDMA ("Ecstasy"). Psychopharmacology 189: 517-530.

364. Quednow BB, Kuhn KU, Hoenig K, Maier W, Wagner M (2004) Prepulse inhibition and habituation of acoustic startle response in male MDMA ('ecstasy') users, cannabis users, and healthy controls. Neuropsychopharmacology 29: 982-990.

365. Quednow BB, Kuhn KU, Stelzenmueller R, Hoenig K, Maier W, et al. (2004) Effects of serotonergic and noradrenergic antidepressants on auditory startle response in patients with major depression. Psychopharmacology 175: 399-406.

366. Quednow BB, Westheide J, Kuhn KU, Werner P, Maier W, et al. (2006) Normal prepulse inhibition and habituation of acoustic startle response in suicidal depressive patients without psychotic symptoms. Journal of Affective Disorders 92: 299-303.

367. Quiske A, Helmstaedter C, Lux S, Elger CE (2000) Depression in patients with temporal lobe epilepsy is related to mesial temporal sclerosis. Epilepsy Research 39: 121-125.

368. Rähmer E (1976) Durchgangssyndrome und Befindlichkeitsstörungen bei chirurgischen und internistischen Patienten [Transition syndromes and affectivity disorders in surgical and internistic patients] [doctoral dissertation]. Nürnberg: University of Erlangen-Nürnberg.

369. Rainov N, Rosendahl W, Hennig H, Burkert W (1993) Neuropsychologische Untersuchungen bei Hypophysenadenompatienten: Versuch einer Verbesserung der kognitiven Parameter mit dem Neuropeptid Desamino-D-Arginin-Vasopressin [Neuropsychological examinations of patients with hypophysisadenoma: Attempt of an improvement of cognitive parameters through the neuropeptide desamino-D-arginine-vasopressine]. Zeitschrift für Medizinische Psychologie 2: 72-81.

370. Rapp AM, Leube DT, Erb M, Grodd W, Kircher TTJ (2004) Neural correlates of metaphor processing. Cognitive Brain Research 20: 395-402.

371. Raschke W (1976) Zum Verhalten von Leistungs- und Intelligenztests bei organischer psychischer Beeinträchtigung: Untersuchungen mit dem Böcker-Test, Benton-Test, Mehrfachwahl-Wortschatz-Test (MWT) und dem Raven-Test [Performance and intelligence tests and organic psychologic impairments: An investigation using the Böcker-test, Benton-test, Multiple-Choice Vocabulary Intelligence-test (MWT), and the Raven-test] [doctoral dissertation]. Nürnberg: University of Erlangen-Nürnberg.

372. Reis O (1997) Risiken und Ressourcen für die Persönlichkeitsentwicklung im Übergang zum Erwachsenenalter [Risks and ressources for development of personality in the transition to adult age]. Weinheim: Psychologie Verlags Union.

373. Reischies FM (1988) Neuropsychologische Befunde bei der Depression im Involutionsalter und Senium und ihre Beziehung zur regionalen Hirndurchblutung [Neuropsychological evidence of depression in the involutionary age and senium and its relationship to regional cranial blood flow]. In: Oepen G, editor. Psychiatrie des rechten und linken Gehirns: Neuropsychologische Ansätze zum Verständnis von „Persönlichkeit“, „Depression“ und „Schizophrenie“ [Psychiatry of the right and left brain: Neuropsychological attempts for the understanding of personality, depression, and schizophrenia]. Köln: Deutscher Ärzte-Verlag. pp. 187-197.

374. Rentrop M, Backenstrass M, Jaentsch B, Kaiser S, Roth A, et al. (2008) Response inhibition in borderline personality disorder: Performance in a Go/Nogo task. Psychopathology 41: 50-57.

375. Reuter B, Herzog E, Endrass T, Kathmann N (2006) Brain potentials indicate poor preparation for action in schizophrenia. Psychophysiology 43: 604-611.

376. Reuter B, Jager M, Bottlender R, Kathmann N (2007) Impaired action control in schizophrenia: The role of volitional saccade initiation. Neuropsychologia 45: 1840-1848.

377. Reuter B, Rakusan L, Kathmanna N (2005) Poor antisaccade performance in schizophrenia: An inhibition deficit? Psychiatry Research 135: 1-10.

378. Revkin SK, Piazza M, Izard V, Zamarian L, Karner E, et al. (2008) Verbal numerosity estimation deficit in the context of spared semantic representation of numbers: A neuropsychological study of a patient with frontal lesions. Neuropsychologia 46: 2463-2475.

379. Richter D, Venzke A, Settelmayer J, Reker T (2002) High rates of inpatient readmissions of alcohol addicted patients - Heavy users or chronically ill patients? Psychiatrische Praxis 29: 364-368.

380. Riedel M, Muller N, Spellmann I, Engel RR, Musil R, et al. (2007) Efficacy of olanzapine versus quetiapine on cognitive dysfunctions in patients with an acute episode of schizophrenia. European Archives of Psychiatry and Clinical Neuroscience 257: 402-412.

381. Riedel M, Spellmann I, Strassnig M, Douhet A, Dehning S, et al. (2007) Effects of risperidone and quetiapine on cognition in patients with schizophrenia and predominantly negative symptoms. European Archives of Psychiatry and Clinical Neuroscience 257: 360-370.

382. Riedel RR, Helmstaedter C, Bulau P, Durwen HF, Brackmann H, et al. (1992) Early signs of cognitive deficits among Human Immunodeficiency Virus-positive hemophiliacs. Acta Psychiatrica Scandinavica 85: 321-326.

383. Riedel R-R, Helmstaedter C, Bülau P, Brackmann H-H, Niese D, et al. (1991) Neuropsychiatrische Untersuchung von 181 HIV-positiven Hämophilen (WR 2-6) [Neuropsychiatric examination of 181 HIV-positive subjects with haemophilia (WR 2-6)]. AIDS-Forschung 6: 70-75.

384. Rief W, Shaw R, Fichter MM (1998) Elevated levels of psychophysiological arousal and cortisol in patients with somatization syndrome. Psychosomatic Medicine 60: 198-203.

385. Roder V (1988) Untersuchungen zur Effektivität kognitiver Therapieinterventionen mit schizophrenen Patienten [Investigation to the effectivity of cognitve therapeutic interventions in schizophrenic patients] [doctoral dissertation]. Bern: Universtiy of Bern.

386. Roder V (1990) Evaluation einer kognitiven Schizophrenietherapie [Evaluation of a cognitive therapy of schizophrenia]. In: Kühne GE, Brenner HD, Huber G, editors. Kognitive Therapie bei Schizophrenen [Cognitive therapy in persons with schizophrenia]. Jena: G. Fischer. pp. 27-39.

387. Rodig G (2002) Memory function in the early postoperative period after cardiac surgery - Impact of the anaesthetic procedure and comparison with memory function after vascular surgery. Anasthesiologie & Intensivmedizin 43: 431-455.

388. Rodig G, Rak A, Kasprzak P, Hobbhahn J (1999) Evaluation of self-reported failures in cognitive function after cardiac and noncardiac surgery. Anaesthesia 54: 826-830.

389. Roesch-Ely D, Scheffel H, Weiland S, Schwaninger M, Hundemer HP, et al. (2005) Differential dopaminergic modulation of executive control in healthy subjects. Psychopharmacology 178: 420-430.

390. Roesch-Ely D, Weiland S, Scheffel H, Schwaninger M, Hundemer HP, et al. (2006) Dopaminergic modulation of semantic priming in healthy volunteers. Biological Psychiatry 60: 604-611.

391. Rollnik JD, Borsutzky M, Huber TJ, Mogk H, Seifert J, et al. (2002) Short-term cognitive improvement in schizophrenics treated with typical and atypical neuroleptics. Neuropsychobiology 45: 74-80.

392. Rosche J, Uhlmann C, Froscher W (2004) On the value of neuropsychological short tests in epileptology. Nervenarzt 75: 1204-1208.

393. Rosche J, Uhlmann C, Nussle S, Froscher W (2001) Neuropsychological screening and inpatient behaviour in patients with epilepsy. Aktuelle Neurologie 28: 455-459.

394. Rosche J, Uhlmann C, Weber R (2003) Influence of age at onset, age and duration of illness on cognitive abilities, in patients with refractory epilepsy. Fortschritte der Neurologie Psychiatrie 71: 595-599.

395. Rosche J, Uhlmann C, Weber R (2004) Changes of coping strategies in patients with therapy refractory epilepsy in the course of a ward based treatment with a holistic therapeutic approach. Psychotherapie Psychosomatik Medizinische Psychologie 54: 4-8.

396. Rosenfeldt B (1991) Kognitive Organisation bei psychotischen Erkrankungen: Leistungsdefizite und ihre diagnostische Bedeutung [Cognitive organization in psychotic disorders: Performance deficits and their diagnostic relevance] [doctoral dissertation]. Bern: University of Bern.

397. Rossner P, Rockstroh B, Cohen R, Wagner M, Elbert T (1999) Event-related potential correlates of proactive interference in schizophrenic patients and controls. Psychophysiology 36: 199-208.

398. Roth F, Lehrl S (1973) Multidimensional self-rating scale for classification of pain. Arzneimittel-Forschung/Drug Research 23: 997-999.

399. Rottig D, Leplow B, Eger K, Ludolph AC, Graf M, et al. (2006) Only subtle cognitive deficits in non-bulbar amyotrophic lateral sclerosis patients. Journal of Neurology 253: 333-339.

400. Rübe S, Rübe I (1992) Entwicklung und Erprobung eines Untersuchungsverfahrens zum Textlernen [Development and test of an examination method for text learning] [doctoral dissertation]. Leipzig: University of Leipzig.

401. Rudolph E (1992) Untersuchung zur intellektuellen Leistungsfähigkeit bei Alkoholkranken und Neurotikern unter besonderer Berücksichtigung der Basiskomponenten der Intelligenz mit Hilfe von Lerntests und computerunterstützten Verfahren [Investigation of the intellectual performance capabilities of alcohol-dependent and neurotic persons considering basic components of intelligence using learning tests and computer-assisted measures] [doctoral dissertation]. Leipzig: University of Leipzig.

402. Rujescu D, Meisenzahl EM, Krejcova S, Giegling I, Zetzsche T, et al. (2007) Plexin B3 is genetically associated with verbal performance and white matter volume in human brain. Molecular Psychiatry 12: 190-194.

403. Rupp CI, Fleischhacker WW, Drexler A, Hausmann A, Hinterhuber H, et al. (2006) Executive function and memory in relation to olfactory deficits in alcohol-dependent patients. Alcoholism-Clinical and Experimental Research 30: 1355-1362.

404. Rupp CI, Fleischhacker WW, Kemmler G, Kremser C, Bilder RM, et al. (2005) Olfactory functions and volumetric measures of orbitofrontal and limbic regions in schizophrenia. Schizophrenia Research 74: 149-161.

405. Rupp CI, Kurz M, Kemmler G, Mair D, Hausmann A, et al. (2003) Reduced olfactory sensitivity, discrimination, and identification in patients with alcohol dependence. Alcoholism-Clinical and Experimental Research 27: 432-439.

406. Rusch N, Weber M, Il'yasovc KA, Lieb K, Ebert D, et al. (2007) Inferior frontal white matter microstructure and patterns of psychopathology in women with borderline personality disorder and comorbid attention-deficit hyperactivity disorder. Neuroimage 35: 738-747.

407. Russ M, Fischer P-A (1989) Reaktionszeit und Aufgabenkomplexität: Der Komplexitätseffekt als ein neuropsychologischer Indikator für den Schweregrad der zerebralen Beeinträchtigung [Reaction time and task complexity: The effect of complexity as a neuropsychological indicator for severeness of cerebral impairments]. Zeitschrift für Differentielle und Diagnostische Psychologie 10: 145-163.

408. Saletu A, Pirker-Fruhauf H, Saletu F, Linzmayer L, Anderer P, et al. (2005) Controlled clinical and psychometric studies on the relation between periodontitis and depressive mood. Journal of Clinical Periodontology 32: 1219-1225.

409. Sartorius A, Ruf M, Kief C, Demirakca T, Bailer J, et al. (2008) Abnormal amygdala activation profile in pedophilia. European Archives of Psychiatry and Clinical Neuroscience 258: 271-277.

410. Sartory G, Zorn C, Groetzinger G, Windgassen K (2005) Computerized cognitive remediation improves verbal learning and processing speed in schizophrenia. Schizophrenia Research 75: 219-223.

411. Satzger W, Hampel H, Padberg F, Burger K, Nolde T, et al. (2001) Practical application of the CERAD test battery in screening for neuropsychological dementia. Nervenarzt 72: 196-203.

412. Sauer H, Hornstein C, Richter P, Mortimer A, Hirsch SR (1999) Symptom dimensions in old-age schizophrenics - Relationship to neuropsychological and motor abnormalities. Schizophrenia Research 39: 31-38.

413. Scheid R, Walther KR, Guthke T, Preul C, von Cramon DY (2006) Cognitive sequelae of diffuse axonal injury. Archives of Neurology 63: 418-424.

414. Scheurich A, Fellgiebel A, Schermuly I, Bauer S, Wolfges R, et al. (2008) Experimental evidence for a motivational origin of cognitive impairment in major depression. Psychological Medicine 38: 237-246.

415. Scheurich A, Muller MJ, Szegedi A, Anghelescu I, Klawe C, et al. (2004) Neuropsychological status of alcohol-dependent patients: Increased performance through goal-setting instructions. Alcohol and Alcoholism 39: 119-125.

416. Schiel R, Bocklitz G, Braun A, Leppert K, Stein G, et al. (2003) Cognitive function and quality of diabetes care in patients with type-2-diabetes mellitus in general practitioner practice. European Journal of Medical Research 8: 419-427.

417. Schiel R, Braun A, Muller R, Helbich C, Siefke S, et al. (2004) A structured treatment and teaching program specially designed for patients with type-2 diabetes mellitus, insulin therapy, and impaired cognitive function (DikoL). Medizinische Klinik 99: 285-292.

418. Schiltz K, Witzel J, Northoff G, Zierhut K, Gubka U, et al. (2007) Brain pathology in pedophilic offenders - Evidence of volume reduction in the right amygdala and related diencephalic structures. Archives of General Psychiatry 64: 737-746.

419. Schlagenhauf F, Juckel G, Koslowski M, Kahnt T, Knutson B, et al. (2008) Reward system activation in schizophrenic patients switched from typical neuroleptics to olanzapine. Psychopharmacology 196: 673-684.

420. Schlagenhauf F, Wuestenberg T, Schmack K, Dinges M, Wrase J, et al. (2008) Switching schizophrenia patients from typical neuroleptics to olanzapine: Effects on BOLD response during attention and working memory. European Neuropsychopharmacology 18: 589-599.

421. Schmidinger M, Linzmayer L, Becherer A, Fazeny-Doerner B, Fakhrai N, et al. (2003) Psychometric- and quality-of-life assessment in long-term glioblastoma survivors. Journal of Neuro-Oncology 63: 55-61.

422. Schmidt H, Schmidt R, Fazekas F, Semmler J, Kapeller P, et al. (1996) Apolipoprotein E e4 allele in the normal elderly: Neuropsychologic and brain MRI correlates. Clinical Genetics 50: 293-299.

423. Schmidt-Atzert L, Buhner M, Enders P (2006) Do concentration tests assess concentration? Analyzing components of concentration test performances. Diagnostica 52: 33-44.

424. Schmitt-Eliassen J, Ferstl R, Wiesner C, Deuschl G, Witt K (2007) Feedback-based versus observational classification learning in healthy aging and Parkinson's disease. Brain Research 1142: 178-188.

425. Schneider C, Fulda S, Schulz H (2004) Daytime variation in performance and tiredness/sleepiness ratings in patients with insomnia, narcolepsy, sleep apnea and normal controls. Journal of Sleep Research 13: 373-383.

426. Schneider-Axmann T, Kamer T, Moroni M, Maric N, Tepest R, et al. (2006) Relation between cerebrospinal fluid, gray matter and white matter changes in families with schizophrenia. Journal of Psychiatric Research 40: 646-655.

427. Schonauer K, Achtergarde D, Reker T (1998) Lipreading in prelingually deaf and hearing patients with schizophrenia. Journal of Nervous and Mental Disease 186: 247-249.

428. Schoning S, Engelien A, Kugel H, Schafer S, Schiffbauer H, et al. (2007) Functional anatomy of visuo-spatial working memory during mental rotation is influenced by sex, menstrual cycle, and sex steroid hormones. Neuropsychologia 45: 3203-3214.

429. Schreiber H, Gaigalat T, Wiedemuth-Catrinescu U, Graf M, Uttner I, et al. (2005) Cognitive function in bulbar- and spinal-onset amyotrophic lateral sclerosis - A longitudinal study in 52 patients. Journal of Neurology 252: 772-781.

430. Schulte RM, Burkard G, Hippe W, Kuntz HD (1986) Die Entwicklung eines Psychodiagnostikums für Gastritis-Patienten: Ergebnisse einer Pilotstudie [Development of a psychodiagnostical measure for patients suffering from gastritis: Results of a pilot-study]. Psycho 4: 950-959.

431. Schultze-Lutter F, Ruhrmann S, Picker H, von Reventlow HG, Daumann B, et al. (2007) Relationship between subjective and objective cognitive function in the early and late prodrome. British Journal of Psychiatry 191: 43-51.

432. Schulz D, Kopp B, Kunkel A, Faiss JH (2006) Cognition in the early stage of multiple sclerosis. Journal of Neurology 253: 1002-1010.

433. Schulze-Rauschenbach SC, Harms U, Schlaepfer TE, Maier W, Falkai P, et al. (2005) Distinctive neurocognitive effects of repetitive transcranial magnetic stimulation and electroconvulsive therapy in major depression. British Journal of Psychiatry 186: 410-416.

434. Schutze C, Bongard I, Marbach S, Brand A, Herzog MH (2007) Collinear contextual suppression in schizophrenic patients. Psychiatry Research 150: 237-243.

435. Schweizer A, Lehmann E, Schreiber M, Heddergott J, Groth J (1998) Cognitive ability and self-evaluated symptoms of aging - a seven year follow-up study with elderly subjects. Aging: 103-112.

436. Schweizer K, Koch W (2001) A revision of Cattell's investment theory: Cognitive properties influencing learning. Learning and Individual Differences 13: 57-82.

437. Seeber A, Schaper M, Zupanic M, Blaszkewicz M, Demes P, et al. (2004) Toluene exposure below 50 ppm and cognitive function: a follow-up study with four repeated measurements in rotogravure printing plants. International Archives of Occupational and Environmental Health 77: 1-9.

438. Seidel I (1992) Kognitive Störungen bei der Drogenabhängigkeit vom Alkohol-(Barbiturat)Typ und ihre Restitution in den ersten vier Abstinenzmonaten [Cognitive impairments in drug-dependence of the alcohol-(barbiturate-)type and their restitution in the first four months of abstinence] [doctoral dissertation]. Leipzig: University of Leipzig.

439. Seifert J, Peters E, Jahn K, Metzner C, Ohlmeier M, et al. (2004) Treatment of alcohol withdrawal: chlormethiazole vs. carbamazepine and the effect on memory performance - A pilot study. Addiction Biology 9: 43-51.

440. Seifert J, Seeland I, Borsutzky M, Passie T, Rollnik JD, et al. (2003) Effects of acute alcohol withdrawal on memory performance in alcohol-dependent patients: A pilot study. Addiction Biology 8: 75-80.

441. Seiferth NY, Pauly K, Habel U, Kellermann T, Shah NJ, et al. (2008) Increased neural response related to neutral faces in individuals at risk for psychosis. NeuroImage 40: 289-297.

442. Seiferth NY, Pauly K, Kellermann T, Shah NJ, Ott G, et al. (2009) Neuronal Correlates of Facial Emotion Discrimination in Early Onset Schizophrenia. Neuropsychopharmacology 34: 477-487.

443. Seitz RJ, Canavan AGM, Yaguez L, Herzog H, Tellmann L, et al. (1997) Representations of graphomotor trajectories in the human parietal cortex: Evidence for controlled processing and automatic performance. European Journal of Neuroscience 9: 378-389.

444. Sheldrick AJ, Krug A, Markov V, Leube D, Michel TM, et al. (2008) Effect of COMT val(158)met genotype on cognition and personality. European Psychiatry 23: 385-389.

445. Shing YL, Werkle-Bergner M, Li SC, Lindenberger U (2008) Associative and strategic components of episodic memory: A life-span dissociation. Journal of Experimental Psychology-General 137: 495-513.

446. Sieg J, Leplow B, Hand I (1999) Neuropsychological deficits and treatment response in obsessive-compulsive disorder. Verhaltenstherapie 9: 7-14.

447. Simon AE, Dvorsky DN, Boesch J, Roth B, Isler E, et al. (2006) Defining subjects at risk for psychosis: A comparison of two approaches. Schizophrenia Research 81: 83-90.

448. Sittinger H, Muller M, Schweizer I, Merkelbach S (2002) Mild cognitive impairment after viral meningitis in adults. Journal of Neurology 249: 554-560.

449. Smith J, Goodnow JJ (1999) Unasked-for support and unsolicited advice: Age and the quality of social experience. Psychology and Aging 14: 108-121.

450. Smole S (1985) Frauenalkoholismus und Rollenbild [Alcoholism in women and role models] [doctoral dissertation]. Vienna: University of Vienna.

451. Spellmann I, Muller N, Musil R, Zill P, Douhet A, et al. (2008) Associations of SNAP-25 polymorphisms with cognitive dysfunctions in Caucasian patients with schizophrenia during a brief trail of treatment with atypical antipsychotics. European Archives of Psychiatry and Clinical Neuroscience 258: 335-344.

452. Spillmann L, Laskowski W, Lange KW, Kasper E, Schmidt D (2000) Stroke-blind for colors, faces and locations: Partial recovery after three years. Restorative Neurology and Neuroscience 17: 89-103.

453. Spitzer C, Haug HJ, Freyberger HJ (1997) Dissociative symptoms in schizophrenic patients with positive and negative symptoms. Psychopathology 30: 67-75.

454. Sprengelmeyer R (1993) Motorische, kognitive und visuell-perzeptive Prozesse bei Chorea Huntington und Morbus Parkinson [Motor, cognitive, and visual-perceptive processes in Chorea Huntington and Morbus Parkinson] [doctoral dissertation]. Bielefeld: University of Bielefeld.

455. Sprengelmeyer R, Young AW, Sprengelmeyer A, Calder AJ, Rowland D, et al. (1997) Recognition of facial expressions: Selective impairment of specific emotions in Huntington's disease. Cognitive Neuropsychology 14: 839-879.

456. Steingass HP, Sartory G, Canavan AGM (1994) Chronic alcoholism and cognitive function - General decline or patterned impairment. Personality and Individual Differences 17: 97-109.

457. Steller U, Beck U (1987) Multiple-sclerosis presenting with symptomatic psychosis - Case-study using NMR and psychological tests. Nervenarzt 58: 256-260.

458. Stetter F, Schoon M, Taubert S, Wegner C, Mann K, et al. (1990) Veränderungsmessungen bei Alkoholabhängigen. Teil II: Faktoren- und Varianzanalyse neuropsychologischer Ergebnisse [Measurement of change in alcohol dependent subjects. Part II: Neuropsychologic evidence of factoranalysis and analysis of variance]. In: Baumann U, Fähndrich E, Stieglitz RD, Woggon B, editors. Veränderungsmessung in Psychiatrie und Klinischer Psychologie: Theoretische, methodische und empirische Beiträge [Measurement of change in psychiatry and clinical psychology: Theoretical, methodical, and empirical contributions]. München: Profil. pp. 119-133.

459. Stevens A, Peschk I, Schwarz J (2007) Implicit learning, executive function and hedonic activity in chronic polydrug abusers, currently abstinent polydrug abusers and controls. Addiction 102: 937-946.

460. Stieglitz RD, Albrecht J, Lundt A, Pittlik V, Hedde HP (1988) Psychopathometrics on patients with HIV infection. Nervenarzt 59: 330-336.

461. Straub R, Straub B, Lehrl S (1976) Ein Kurztest zur Quantifizierung der zyklothymen Depression [A short test for quantification of cyclothymic depression]. Psychopathometrie 2: 129-136.

462. Strohle A, Stoy M, Wrase J, Schwarzer S, Schlagenhauf F, et al. (2008) Reward anticipation and outcomes in adult males with attention-deficit/hyperactivity disorder. Neuroimage 39: 966-972.

463. Suslow T, Dannlowski U, Lalee-Mentzel J, Donges US, Arolt V, et al. (2004) Spatial processing of facial emotion in patients with unipolar depression: a longitudinal study. Journal of Affective Disorders 83: 59-63.

464. Suslow T, Junghanns K, Arolt V (2001) Detection of facial expressions of emotions in depression. Perceptual and Motor Skills 92: 857-868.

465. Suslow T, Junghanns K, Donges US, Arolt V (2001) Alexithymia and automatic processing of verbal and facial affect stimuli. Cahiers de Psychologie Cognitive-Current Psychology of Cognition 20: 297-324.

466. Suslow T, Schonauer K, Ohrmann P, Eikelmann B, Reker T (2000) Prediction of work performance by clinical symptoms and cognitive skills in schizophrenic outpatients. Journal of Nervous and Mental Disease 188: 116-118.

467. Tabeling S, Kopp B, Braun M, Moschner C, Wessel K (2007) Crossed aphasia or dysexecutive syndrome? A case report. Fortschritte der Neurologie Psychiatrie 75: 484-489.

468. Teigeler R, Pieprczyk L (1983) Unterscheiden sich Intelligenzmessungen per Hand und per Computer? [Is there a difference between personal- or computer-administrated measurement of intelligence?]. Grundlagenstudien aus Kybernetik und Geisteswissenschaft 24: 115-121.

469. Tesar N, Bandion K, Baumhackl U (2005) Efficacy of a neuropsychological training programme for patients with multiple sclerosis - A randomised controlled trial. Wiener Klinische Wochenschrift 117: 747-754.

470. Tesch-Romer C (1997) Psychological effects of hearing aid use in older adults. Journals of Gerontology Series B-Psychological Sciences and Social Sciences 52: 127-138.

471. Tetzlaff K, Leplow B, ten Thoren C, Dahme B (1999) Perception of dyspnea during histamine- and methacholine-induced bronchoconstriction. Respiration 66: 427-433.

472. Thomasius R, Petersen K, Buchert R, Andresen B, Zapletalova P, et al. (2003) Mood, cognition and serotonin transporter availability in current and former ecstasy (MDMA) users. Psychopharmacology 167: 85-96.

473. Thone AIT, Zysset S, von Cramon DY (1999) Retrieval of long-term memory in patients with brain injuries. Journal of Clinical and Experimental Neuropsychology 21: 798-815.

474. Titz C, Behrendt J, Menge U, Hasselhorn M (2008) A reassessment of negative priming within the inhibition framework of cognitive aging: There is more in it than previously believed. Experimental Aging Research 34: 340-366.

475. Trenckmann U, Briese R, Adolph H-M, Schlebusch P (1999) Rückfall-Früherkennung: Evaluation eines semistandardisierenden Trainingsprogramms für schizophren gefährdete Menschen zur verbesserten Selbstwahrnehmung eines drohenden Rezidivs [Early diagnostics of relapses: Evaluation of a semi-standardized training program for schizophrenia risk-groups for enhanced self-perception of an imminent recurrence]. In: Lasar M, Ribbert H, editors. Kognitive und motivationale Prozesse bei schizophrener Erkrankung [Cognitive and motivational processes in schizophrenic disorders]. Regensburg: Roderer. pp. 54-66.

476. Triebig G, Claus D, Csuzda I, Druschky KF, Holler P, et al. (1988) Cross-sectional epidemiological study on neurotoxicity of solvents in paints and lacquers. International Archives of Occupational and Environmental Health 60: 233-241.

477. Triebig G, Grobe T, Saure E, Schaller KH, Weltle D, et al. (1984) Investigations on neurotoxicity of chemical substances at the workplace. 6. longitudinal study in persons occupationally exposed to mercury. International Archives of Occupational and Environmental Health 55: 19-31.

478. Triebig G, Lehrl S, Weltle D, Schaller K-H, Valentin H. Arbeitsmedizinische und testpsychologische Feldstudie zur akuten und chronischen Neurotoxizität von Styrol unter gegenwärtigen Expositionsbedingungen [Occupational medical and testpsychological field-study on acute and chronic neurotoxicity of styrol in current expositional conditions]; 1988; Innsbruck, 05.05.1988. pp. 93-97.

479. Tucha L, Tucha O, Laufkotter R, Walitza S, Klein HE, et al. (2008) Neuropsychological assessment of attention in adults with different subtypes of attention-deficit/hyperactivity disorder. Journal of Neural Transmission 115: 269-278.

480. Tucha O, Mecklinger L, Laufkotter R, Klein HE, Walitza S, et al. (2006) Methylphenidate-induced improvements of various measures of attention in adults with Attention Deficit Hyperactivity Disorder. Journal of Neural Transmission 113: 1575-1592.

481. Ueber R (1998) Working memory und Schizophrenie: Eine Untersuchung zu spezifischen Gedächtnisdefiziten schizophrener Patienten in Abhängigkeit ohres Symptomprofils [Working memory and schizophrenia: An investigation of specific memory deficits of schizophrenic patients depending on their symptom profile]. Regensburg: Roderer.

482. Ullsperger M, Mecklinger A, Matthes-von Cramon G, von Cramon DY (2000) Transient global ischemia specifically modulates visual P300 scalp distribution. Clinical Neurophysiology 111: 2245-2254.

483. Unterrainer JM, Rahm B, Leonhart R, Ruff CC, Halsband U (2003) The Tower of London: the impact of instructions, cueing, and learning on planning abilities. Cognitive Brain Research 17: 675-683.

484. Uttner I, Wahllander-Danek U, Danek A (2003) Cognitive impairment in adults with neurofibromatosis type 1. Fortschritte der Neurologie Psychiatrie 71: 157-162.

485. Valerius G, Lumpp A, Kuelz AK, Freyer T, Voderholzer U (2008) Reversal learning as a neuropsychological indicator for the neuropathology of obsessive compulsive disorder? A behavioral study. Journal of Neuropsychiatry and Clinical Neurosciences 20: 210-218.

486. van Thriel C, Kleinsorge T, Zupanic M, Seeber A (2000) Switching attention-additional aspects for the analysis. Neurotoxicology 21: 795-804.

487. van Thriel C, Zupanic M, Sietmann B, Demes P, Willer H, et al. (1998) Association of biochemical and subjective indicators of drinking habits with performance on different neurobehavioral tasks. Neurotoxicology 19: 713-720.

488. Vauth R, Barth A, Stieglitz RD (2001) Evaluation of a cognitive strategy training in vocational rehabilitation of schizophrenic outpatients. Zeitschrift fur Klinische Psychologie und Psychotherapie 30: 251-258.

489. Vauth R, Corrigan PW, Clauss M, Dietl M, Dreher-Rudolph M, et al. (2005) Cognitive strategies versus self-management skills as adjunct to vocational rehabilitation. Schizophrenia Bulletin 31: 55-66.

490. Viaene M, Veulemans H, Masschelein R (1998) Experience with a vocabulary test for workers previously and still exposed to styrene. Scandinavian Journal of Work Environment & Health 24: 308-311.

491. Vielhaber S, Jakubiczka S, Gaul C, Schoenfeld MA, Debska-Vielhaber G, et al. (2006) Brain H-1 magnetic resonance spectroscopic differences in myotonic dystrophy type 2 and type 1. Muscle & Nerve 34: 145-152.

492. Visani P, Schmutzhard E, Trinka E, Pfausler B, Benke T (2006) Subcortical deficit pattern after brain abscess: a neuropsychological study. European Journal of Neurology 13: 599-603.

493. Vitouch O, Bauer H, Gittler G, Leodolter M, Leodolter U (1997) Cortical activity of good and poor spatial test performers during spatial and verbal processing studied with Slow Potential Topography. International Journal of Psychophysiology 27: 183-199.

494. von Cramon D, Kuhnlein J, Wolfram A (1981) Thalamic dementia. Fortschritte der Neurologie Psychiatrie 49: 129-135.

495. von Giesen HJ, Antke C, Hefter H, Wenserski F, Seitz RJ, et al. (2000) Potential time course of human immunodeficiency virus type 1-associated minor motor deficits - Electrophysiologic and positron emission tomography findings. Archives of Neurology 57: 1601-1607.

496. von Giesen HJ, Backer R, Hefter H, Arendt G (2001) Depression does not influence basal ganglia-mediated psychomotor speed in HIV-1 infection. Journal of Neuropsychiatry and Clinical Neurosciences 13: 88-94.

497. von Giesen HJ, Haslinger BA, Rohe S, Koller H, Arendt G (2005) HIV dementia scale and psychomotor slowing - The best methods in screening for neuro-AIDS. Journal of Neuropsychiatry and Clinical Neurosciences 17: 185-191.

498. von Giesen HJ, Heintges T, Abbasi-Boroudjeni N, Kucukkoylu S, Koller H, et al. (2004) Psychomotor slowing in hepatitis C and HIV infection. Jaids-Journal of Acquired Immune Deficiency Syndromes 35: 131-137.

499. Voracek M, Egle J, Schleicher S, Loibl LM, Sonneck G (2007) The Beliefs in the Inheritance of Risk Factors for Suicide Scale (BIRFSS): Further results on demographic correlates, dimensionality, reliability, and validity. Omega-Journal of Death and Dying 55: 279-296.

500. Wagner M, Baving L, Berg P, Cohen R, Rockstroh B (2006) An ERP investigation of semantic priming, repetition priming, and negative priming in schizophrenic patients. Journal of Psychophysiology 20: 195-211.

501. Weess H-G (1996) Leistungserfassung beim obstruktiven Schlaf-Apnoe-Syndrom: Aufmerksamkeitsbezogene Einschränkungen und deren Reversibilität [Performance assessment and the obstructive sleep-apnoe-syndrome: Impairments and their reversabilities regarding attention]. Regensburg: Roderer.

502. Weidenhammer W, Engel R (1988) Psychometrie und Elektroenzephalographie bei gesunden Hochbetagten [Psychometrics and electronecephalography in healthy adults in old age]. Zeitschrift für Gerontopsychologie und -psychiatrie 1: 105-115.

503. Weidenhammer W, Fischer B, Lehrl S (1986) Erfahrungen mit der kombinierten Therapie aus Antihypoxidotikum und zerebralem Training bei Patienten mit zerebrovaskulärer Insuffizienz [Experiences with a combined therapy of antihypoxidoticum and cerebral training in patients suffering from cerebrovascular insufficiency]. Geriatrics, Pregeriatrics, Rehabilitation 2: 113-116.

504. Weijers HG, Wiesbeck GA, Boning J (2001) Reflection-impulsivity, personality and performance: a psychometric and validity study of the Matching Familiar Figures Test in detoxified alcoholics. Personality and Individual Differences 31: 731-754.

505. Weiss EM, Kemmler G, Deisenhammer EA, Fleischhacker WW, Delazer M (2003) Sex differences in cognitive functions. Personality and Individual Differences 35: 863-875.

506. Weiss EM, Ragland JD, Brensinger CM, Bilker WB, Deisenhammer EA, et al. (2006) Sex differences in clustering and switching in verbal fluency tasks. Journal of the International Neuropsychological Society 12: 502-509.

507. Weiss PH, Fink GR (2009) Grapheme-colour synaesthetes show increased grey matter volumes of parietal and fusiform cortex. Brain 132: 65-70.

508. Weiss PH, Zilles K, Fink GR (2005) When visual perception causes feeling: Enhanced cross-modal processing in grapheme-color synesthesia. NeuroImage 28: 859-868.

509. Welzel G, Fleckenstein K, Mai SK, Hermann B, Kraus-Tiefenbacher U, et al. (2008) Akute neurokognitive Beeintrachtigungen wahrend Radiotherapie bei Patienten mit ZNS-Tumoren. Strahlentherapie und Onkologie 184: 647-654.

510. Welzel G, Fleckenstein K, Schaefer J, Hermann B, Kraus-Tiefenbacher U, et al. (2008) Memory function before and after whole brain radiotherapy in patients with and without brain metastases. International Journal of Radiation Oncology Biology Physics 72: 1311-1318.

511. Wenderlein JM (1976) Wie beurteilen Frauen ihr Wissen über Kontrazeption? (Eine Untersuchung an 386 Frauen unter psychologisch-soziologischen Aspekten [How judge women their knowledge of contraception? (An investigation on 386 women considering psychologic-sociologic aspects)]. Therapeutische Umschau 33: 210-213.

512. Wenderlein JM (1978) IQ in gynecology. Geburtshilfe und Frauenheilkunde 38: 619-628.

513. Werheid K, Koch I, Reichert K, Brass M (2007) Impaired self-initiated task preparation during task switching in Parkinson's disease. Neuropsychologia 45: 273-281.

514. Westheide J, Quednow BB, Kuhn KU, Hoppe C, Cooper-Mahkorn D, et al. (2008) Executive performance of depressed suicide attempters: the role of suicidal ideation. European Archives of Psychiatry and Clinical Neuroscience 258: 414-421.

515. Westheide J, Wagner M, Quednow BB, Hoppe C, Cooper-Mahkorn D, et al. (2007) Neuropsychological performance in partly remitted unipolar depressive patients: focus on executive functioning. European Archives of Psychiatry and Clinical Neuroscience 257: 389-395.

516. Weyer G, Ihl R, Mohs RC, Schambach M, Denkel A, et al. (1993) Validierungsuntersuchungen zu einer deutschen Version der Alzheimer’s Disease Assessment Scale ADAS [Investigations to the validity of a German version of the Alzheimer’s Disease Assessment Scale ADAS]. Zeitschrift für Gerontopsychologie und -psychiatrie 6: 67-81.

517. Wiedl KH, Schottke H, Green MF, Nuechterlein KH (2004) Dynamic testing in schizophrenia: Does training change the construct validity of a test? Schizophrenia Bulletin 30: 703-711.

518. Wiedl KH, Wienobst J, Schottke HH, Green MF, Nuechterlein KH (2001) Attentional characteristics of schizophrenia patients differing in learning proficiency on the Wisconsin Card Sorting Test. Schizophrenia Bulletin 27: 687-695.

519. Wiessner B, Felber W (1981) Agreement between 2 diagnostic intelligence test procedures (HAWIE and MWT-B) in a sample of patients with pronounced psychopathology. Psychiatrie, Neurologie und Medizinische Psychologie 33: 744-748.

520. Wiest G, Lehner-Baumgartner E, Baumgartner C (2006) Panic attacks in an individual with bilateral selective lesions of the amygdala. Archives of Neurology 63: 1798-1801.

521. Wild-Wall N, Willemssen R, Falkenstein M, Beste C (2008) Time estimation in healthy ageing and neurodegenerative basal ganglia disorders. Neuroscience Letters 442: 34-38.

522. Willemssen R, Muller T, Schwarz M, Hohnsbein J, Falkenstein M (2008) Error processing in patients with Parkinson's disease: the influence of medication state. Journal of Neural Transmission 115: 461-468.

523. Windischberger C, Lamm C, Bauer H, Moser E (2002) Consistency of inter-trial activation using single-trial fMRI: assessment of regional differences. Cognitive Brain Research 13: 129-138.

524. Windmann S, Wehrmann M, Calabrese P, Gunturkun O (2006) Role of the prefrontal cortex in attentional control over bistable vision. Journal of Cognitive Neuroscience 18: 456-471.

525. Witt K, Borsch K, Daniels C, Walluscheck K, Alfke K, et al. (2007) Neuropsychological consequences of endarterectomy and endovascular angioplasty with stent placement for treatment of symptomatic carotid stenosis - A prospective randomised study. Journal of Neurology 254: 1524-1532.

526. Witt K, Daniels C, Daniel V, Schmitt-Eliassen J, Volkmann J, et al. (2006) Patients with Parkinson's disease learn to control complex systems - an indication for intact implicit cognitive skill learning. Neuropsychologia 44: 2445-2451.

527. Witt K, Daniels C, Schmitt-Eliassen J, Kernbichler J, Rehm S, et al. (2006) The impact of normal aging and Parkinson's disease on response preparation in task-switching behavior. Brain Research 1114: 173-182.

528. Witt K, Nuhsman A, Deuschl G (2002) Dissociation of habit-learning in Parkinson's and cerebellar disease. Journal of Cognitive Neuroscience 14: 493-499.

529. Witt K, Nuhsman A, Deuschl G (2002) Intact artificial grammar learning in patients with cerebellar degeneration and advanced Parkinson's disease. Neuropsychologia 40: 1534-1540.

530. Wittorf A, Wiedemann G, Buchkremer G, Klingberg S (2007) Prediction of community outcome in schizophrenia 1 year after discharge from inpatient treatment. European Archives of Psychiatry and Clinical Neuroscience 258: 48-58.

531. Wobrock T, Kamer T, Roy A, Vogeley K, Schneider-Axmann T, et al. (2008) Reduction of the internal capsule in families affected with schizophrenia. Biological Psychiatry 63: 65-71.

532. Wobrock T, Sittinger H, Behrendt B, D'Amelio R, Falkai P, et al. (2007) Comorbid substance abuse and neurocognitive function in recent-onset schizophrenia. European Archives of Psychiatry and Clinical Neuroscience 257: 203-210.

533. Wolfradt U, Felfe J, Köster T (2001) Self-perceived emotional intelligence and creative personality. Imagination, Cognition and Personality 21: 293-309.

534. Wolwer W, Brinkmeyer J, Riesbeck M, Freimuller L, Klimke A, et al. (2008) Neuropsychological impairments predict the clinical course in schizophrenia. European Archives of Psychiatry and Clinical Neuroscience 258: 28-34.

535. Yaguez L, Canavan AGM, Lange HW, Homberg V (1999) Motor learning by imagery is differentially affected in Parkinson's and Huntington's diseases. Behavioural Brain Research 102: 115-127.

536. Yaguez L, Lange H, Homberg V (2006) Differential effect of Huntington's and Parkinson's diseases in programming motor sequences of varied lengths. Journal of Neurology 253: 186-193.

537. Yaguez L, Nagel D, Hoffman H, Canavan AGM, Wist E, et al. (1998) A mental route to motor learning: Improving trajectorial kinematics through imagery training. Behavioural Brain Research 90: 95-106.

538. Zamarian L, Karner E, Benke T, Donnemiller E, Delazer M (2006) Knowing 7 x 8, but not the meaning of 'elephant': Evidence for the dissociation between numerical and non-numerical semantic knowledge. Neuropsychologia 44: 1708-1723.

539. Zamarian L, Sinz H, Bonatti E, Gamboz N, Delazer M (2008) Normal aging affects decisions under ambiguity, but not decisions under risk. Neuropsychology 22: 645-657.

540. Zamarian L, Visani P, Delazer M, Seppi K, Mair KJ, et al. (2006) Parkinson's disease and arithmetics: The role of executive functions. Journal of the Neurological Sciences 248: 124-130.

541. Zeintl M, Kliegel M (2007) How do verbal distractors influence age-related operation span performance? A manipulation of inhibitory control demands. Experimental Aging Research 33: 163-175.

542. Zeintl M, Kliegel M (2007) The role of inhibitory control in age-related operation span performance. European Journal of Ageing 4: 213-217.

543. Zellner M, Bauml KH (2006) Inhibitory deficits in older adults: List-method directed forgetting revisited. Journal of Experimental Psychology-Learning Memory and Cognition 32: 290-300.

544. Zimber A, Neuhauser T (1995) Alltagsauffälligkeiten bei hirnorganischen Patienten mit unterschiedlichen Schweregraden kognitiver Beeinträchtigung [Everyday conspiciousnesses in cranial organic patients considering differing severities of cognitive impairments]. Zeitschrift für Gerontopsychologie und -psychiatrie 8: 99-114.

545. Zimprich D, Martin M, Kliegel M, Dellenbach M, Rast P, et al. (2008) Cognitive abilities in old age: Results from the Zurich Longitudinal Study on cognitive aging. Swiss Journal of Psychology 67: 177-195.

546. Zorn P, Roder V, Muller DR, Tschacher W, Thommen M (2007) Schema focused emotive behavioural therapy ('SET'): A randomised controlled trial on patients with cluster B and C personality disorders. Verhaltenstherapie 17: 233-241.

547. Zorn P, Roder V, Soravia L, Tschacher W (2008) Evaluation of the "Schema-focused Emotive Behavioural Therapy" (SET) for Patients with Personality Disorders: Results of a Randomised Controlled Trial. Psychotherapie Psychosomatik Medizinische Psychologie 58: 371-378.
